# Site-selective formation of an iron(iv)–oxo species at the more electron-rich iron atom of heteroleptic μ-nitrido diiron phthalocyanines†
†Electronic supplementary information (ESI) available: Experimental and computational details, Cartesian coordinates, characterization data, and ESI-MS spectra. See DOI: 10.1039/c5sc01811k


**DOI:** 10.1039/c5sc01811k

**Published:** 2015-06-16

**Authors:** Ümit İşci, Abayomi S. Faponle, Pavel Afanasiev, Florian Albrieux, Valérie Briois, Vefa Ahsen, Fabienne Dumoulin, Alexander B. Sorokin, Sam P. de Visser

**Affiliations:** a Gebze Technical University , Department of Chemistry , P.O. Box 141, Gebze , 41400 Kocaeli , Turkey . Email: fdumoulin@gtu.edu.tr; b Manchester Institute of Biotechnology and School of Chemical Engineering and Analytical Science , The University of Manchester , 131 Princess Street , Manchester M1 7DN , UK . Email: sam.devisser@manchester.ac.uk; c Institut de Recherches sur la Catalyse et l'Environnement de Lyon (IRCELYON) , UMR 5256 , CNRS-Université Lyon 1 , 2, av. A. Einstein , 69626 Villeurbanne Cedex , France . Email: alexander.sorokin@ircelyon.univ-lyon1.fr; d Centre Commun de Spectrométrie de Masse UMR 5246 , CNRS-Université Claude Bernard Lyon 1 , Université de Lyon , Bâtiment Curien , 43, bd du 11 Novembre , 69622 Villeurbanne Cedex , France; e Synchrotron Soleil , L'orme des merisiers, St-Aubin , 91192 Gif-sur-Yvette , France

## Abstract

Iron(iv)–oxo species have been identified as the active intermediates in key enzymatic processes, and their catalytic properties are strongly affected by the equatorial and axial ligands bound to the metal, but details of these effects are still unresolved. In our aim to create better and more efficient oxidants of H-atom abstraction reactions, we have investigated a unique heteroleptic diiron phthalocyanine complex. We propose a novel intramolecular approach to determine the structural features that govern the catalytic activity of iron(iv)–oxo sites. Heteroleptic μ-nitrido diiron phthalocyanine complexes having an unsubstituted phthalocyanine (Pc**1**) and a phthalocyanine ligand substituted with electron-withdrawing alkylsulfonyl groups (Pc**SO_2_R**) were prepared and characterized. A reaction with terminal oxidants gives two isomeric iron(iv)–oxo and iron(iii)–hydroperoxo species with abundances dependent on the equatorial ligand. Cryospray ionization mass spectrometry (CSI-MS) characterized both hydroperoxo and diiron oxo species in the presence of H_2_O_2_. When *m*-CPBA was used as the oxidant, the formation of diiron oxo species (Pc**SO_2_R**)FeNFe(Pc**1**)=O was also evidenced. Sufficient amounts of these transient species were trapped in the quadrupole region of the mass-spectrometer and underwent a CID-MS/MS fragmentation. Analyses of fragmentation patterns indicated a preferential formation of hydroperoxo and oxo moieties at more electron-rich iron sites of both heteroleptic μ-nitrido complexes. DFT calculations show that both isomers are close in energy. However, the analysis of the iron(iii)–hydroperoxo bond strength reveals major differences for the (Pc**1**)FeN(Pc**SO_2_R**)Fe^III^OOH system as compared to (Pc**SO_2_R**)FeN(Pc**1**)Fe^III^OOH system, and, hence binding of a terminal oxidant will be preferentially on more electron-rich sides. Subsequent kinetics studies showed that these oxidants are able to even oxidize methane to formic acid efficiently.

## Abstract 2

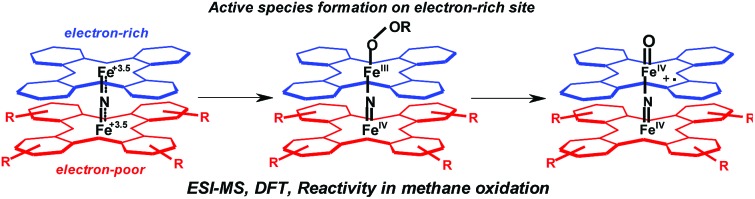
A combination of MS and computation on μ-nitrido bridged diiron complexes reveals H_2_O_2_ binding to the complex and generates an oxidant capable of oxidizing methane.

## Introduction

High-valent iron(iv)–oxo species are key intermediates in biology and involved in essential oxidation processes in Nature.^1^ Metalloenzymes often include an iron(iv)–oxo active species in their catalytic cycle, however, large variations in chemical reactivity and biochemical properties are observed due to the iron coordination environment. For instance, iron(iv)–oxo intermediates contribute as the active oxidant in heme monoxygenases, like the cytochromes P450, whereas in the structurally analogous peroxidases the detoxification of hydrogen peroxide to water is catalyzed.^2^ By contrast, non-heme iron dioxygenases utilize an iron(iv)–oxo active species in their catalytic cycle to transfer an oxygen atom to substrates with functions ranging from DNA base repair and the biosynthesis of antibiotics.^3^ Why nature has this large variety of metalloenzymes that are structurally and functionally diverse is an important question that remains the key focus of extensive research. The differences in chemical reactivity between heme and non-heme iron(iv)–oxo species have been identified as originating from the metal-substrate orbital-interactions and the electron-transfer pathways during the chemical reaction.^4^ In order to understand the sophisticated chemistry of iron(iv)–oxo intermediates in chemistry and biology, many studies have been devoted to the preparation and spectroscopic characterization of these short-lived species using enzymes^5^ as well as model complexes.^6^−^9^ A great variety of mononuclear high-valent iron(iv)–oxo species on the porphyrin,^6^ corrole,^7^ phthalocyanine,^8^ and non-heme ligand^9^ platforms have been prepared at low temperatures and characterized. These studies have given detailed insight into the mechanisms of oxygen atom transfer under difficult catalytic conditions, which in Nature happens under mild conditions. The studies have led to the development of series of bio-inspired chemical systems for selective oxidations, mostly based on mononuclear iron complexes.^10^

Until recently, the research involving binuclear complexes was mainly focused on non-heme diiron, due to its relationship to the enzyme soluble methane monooxygenase,^11^ although some studies have been reported on oxo and hydroxo bridged diiron porphyrin complexes.^12^ One particular ligand system for iron that has gained increasing attention in recent years are those based on phthalocyanine ligands, which specifically showed high reactivity in oxidation reactions under mild conditions.^13^

**Scheme 1:**
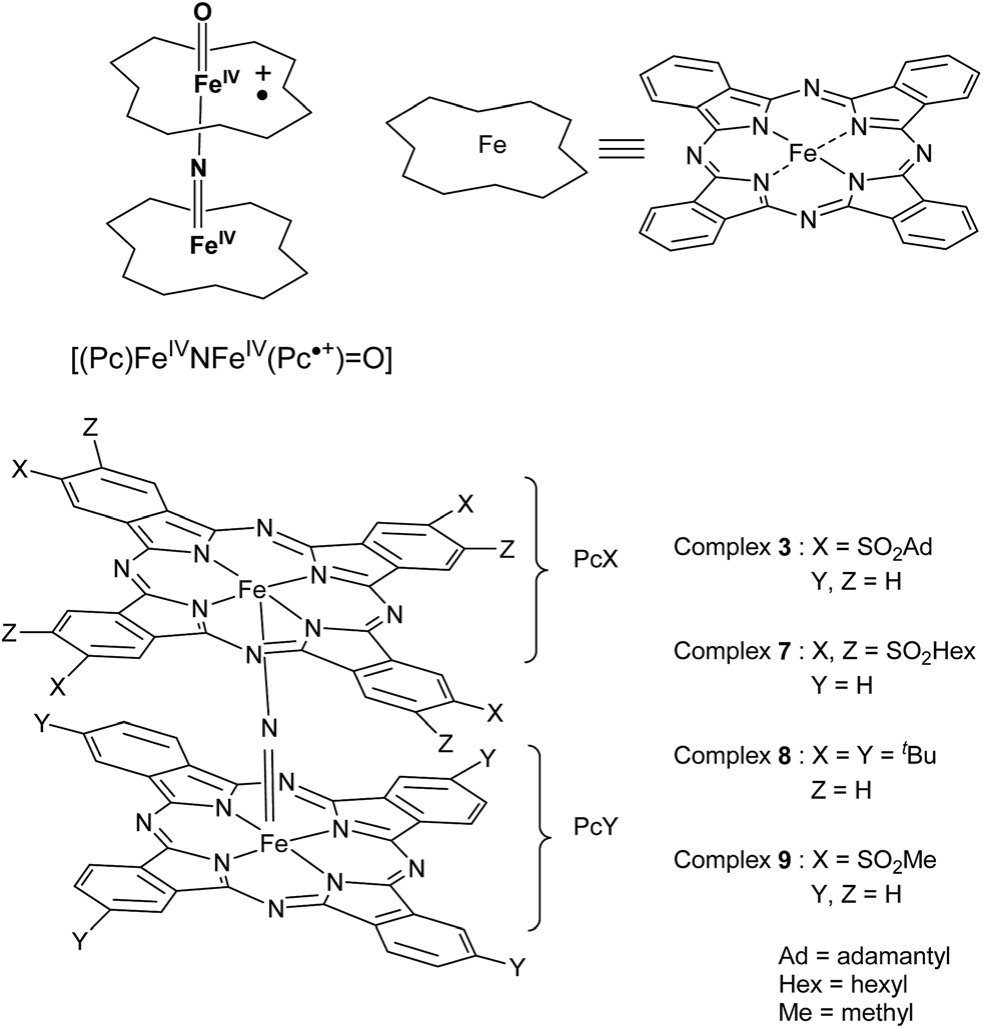
Iron(iv)–oxo species of μ-nitrido diiron phthalocyanine complexes (top) and structures of the complexes used in this work (bottom).

Thus, μ-nitrido diiron phthalocyanines ([(Pc)FeNFe(Pc)], Scheme 1), show surprising catalytic properties, and were found to be able to hydroxylate methane and benzene at near-ambient conditions,^14^ but also defluorinate perfluorinated compounds under oxidative conditions.14*d* None of these reactions are native to the cytochrome P450 enzymes.

μ-Nitrido diiron phthalocyanines belong to the class of stable binuclear single atom bridged complexes that are known to bind metal ions in high oxidation states,^15^ although the nature of the reactivity patterns was determined on whether electron-withdrawing or electron-donating substituents on the periphery of the phthalocyanine moieties were added.^16^,^17^ These complexes were shown to give a large versatility of chemical and catalytic properties and can adopt Fe^III^Fe^IV^ and Fe^IV^Fe^IV^ oxidation states.^18^−^20^ The μ-nitrido diiron macrocyclic complexes were found to be able to activate oxygen-atom donors, such as H_2_O_2_ and *m*-chloroperbenzoic acid (*m*-CPBA), to form a high-valent diiron–oxo species, *i.e.* [(Pc)Fe^IV^NFe^IV^(Pc^+^˙)=O]. The addition of H_2_O_2_ to μ-nitrido diiron tetra-*t*-butylphthalocyanine **8**, [(Pc^*t*Bu^)FeNFe(Pc^*t*Bu^)] (Scheme 1), resulted in the formation of the hydroperoxo complex, [(Pc^*t*Bu^)Fe^IV^NFe^III^(Pc^*t*Bu^)–OOH], followed by the generation of the diiron–oxo species, [(Pc^*t*Bu^)Fe^IV^NFe^IV^(Pc^*t*Bu+^˙)=O], as identified by electrospray ionization-mass spectrometry (ESI-MS) using labelled H_2_^18^O_2_.14*a* With the tetraphenylporphyrin (TPP) platform, we were also able to prepare the corresponding oxo complex [(TPP)Fe^IV^NFe^IV^(TPP^+^˙)=O] with *m*-CPBA oxidant at –90 °C, and characterize it by ESI-MS, low-temperature UV-vis, electron paramagnetic resonance (EPR) and Mössbauer spectroscopic techniques.^21^ It should be noted that high-valent diiron oxo species on the phthalocyanine and porphyrin platforms show superior oxidizing properties compared with mononuclear counterparts, but the origin of these reactivity differences is still poorly understood.

The macrocyclic μ-nitrido diiron phthalocyanine provides a unique possibility to probe the differences in chemical properties of the two iron centers within the same molecule. The question, therefore, is: How do the functional properties of the iron(iv)–nitrido group affect the catalytic functions of the iron(iv)–oxo moiety and how can these be modified? In order to answer these questions, we decided to synthesize localized structures with non-equivalent iron sites, where each of the iron atoms is placed into macrocyclic ligands with very different electronic properties. Our approach opens interesting possibilities but also leads to deeper insight into the formation of the active species responsible for the catalysis. In particular, since such complexes with localized structure have two possible sites where an iron(iv)–oxo species can be formed. Therefore, we designed unique chemical complexes that are based on the same phthalocyanine ligand structure, but exhibiting different electronic properties due to different substitution patterns. In particular, we created structures with one macrocyclic ligand with an electron-poor phthalocyanine group with four or eight alkylsulfonyl substituents, whereas the other macrocyclic ligand contained a more electron-rich unsubstituted phthalocyanine macrocycle. We report here the first synthesis and characterization of this series of unique heteroleptic structures (**3** and **7**, Scheme 1), with alkylsulfonyl electron-withdrawing groups at the periphery of the Pc moiety. In addition, computational modelling was performed on the abbreviated system **9** with either oxo (**9=O**) or hydroperoxo (**9–OOH**) bound. The studies show that the electron-withdrawing character of the equatorial ligand, *i.e.* the phthalocyanine moiety, affects the strength of the iron(iii)–hydroperoxo bond, and, thereby determines the site where the oxo group will bind. The work reveals the subtleties of ligand binding on the chemical and ultimately catalytic properties of metal–oxo complexes. The ESI-MS and DFT results suggest the higher catalytic activity for more electron-rich iron sites. This prediction was confirmed in the catalytic heterogeneous oxidation of methane with H_2_O_2_ using three μ-nitrido diiron phthalocyanine complexes with different substitution patterns.

## Results and discussion

### Synthesis and characterization of diiron complexes

Tetrapyrrolic ligands, such as porphyrin and phthalocyanine, can be used to form dimeric complexes.^18^ Heteroleptic complexes of μ-nitrido tetrapyrroles can be formed of two chemically different ligands. It should be emphasized that the iron sites of the Fe(iii)Fe(iv) unit are not necessarily inequivalent in terms of the oxidation state,^18^,^22^ because the influence of the different ligand field strength of the two macrocycles on iron ions is overcome by the mediated action of the nitrogen bridging atom.22*b* Alternatively, heteroleptic complexes based on the same tetrapyrrolic ligand type but with different substitution patterns, *i.e.* with different electronic properties,^18^ can provide access to localized structure with two different iron sites. Heteroleptic structures with different ligand features can be prepared by initially making monomeric iron(iii) complexes, which are then mixed in a solution with sodium azide and α-chloronaphthalene to form the μ-nitrido diiron complexes.

**Scheme 2:**
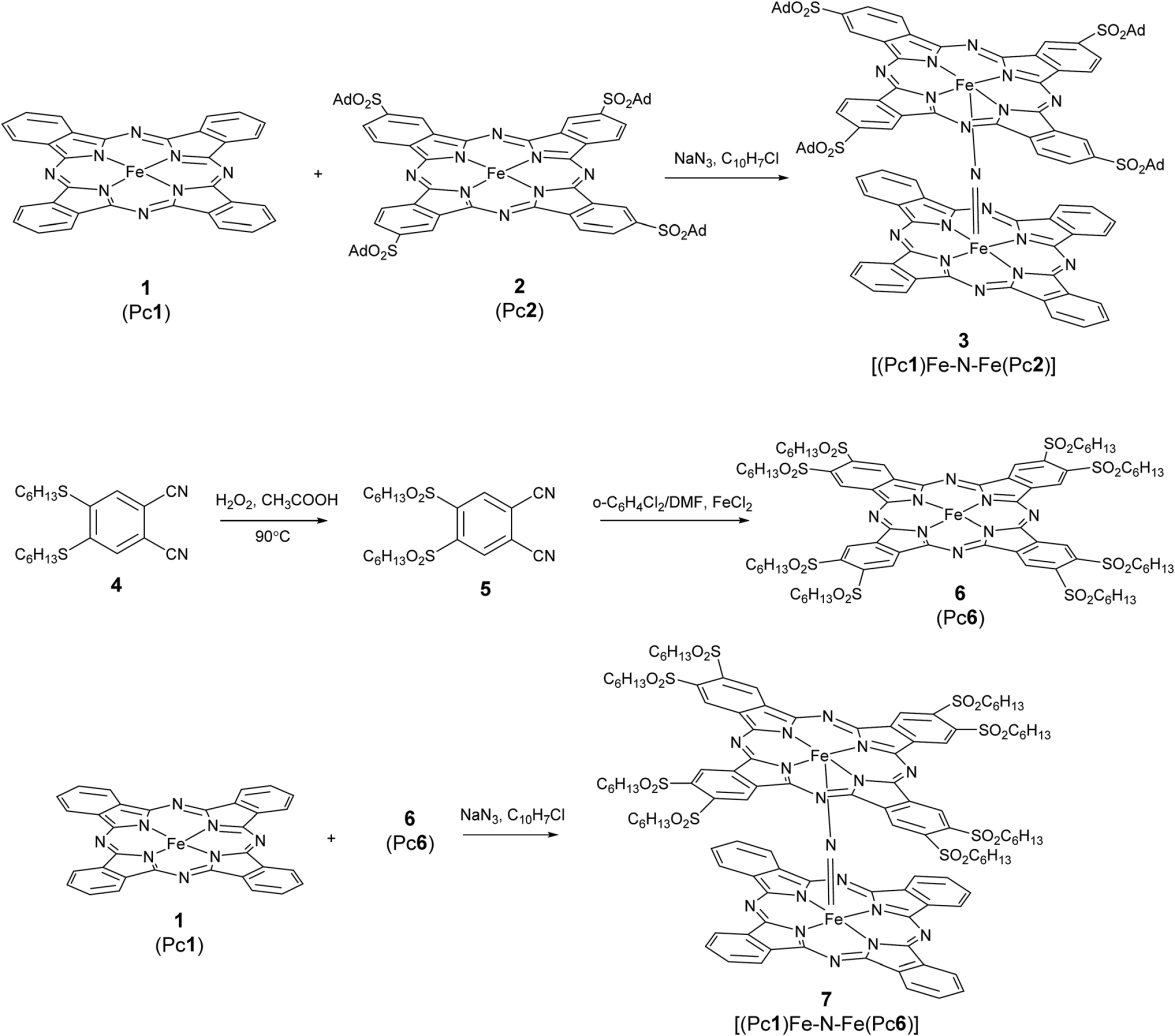
Preparation of heteroleptic μ-nitrido alkylsulfonyl phthalocyaninato iron(ii) **3** and **7**.

The synthesis of alkylsulfonyl substituted phthalonitriles (Scheme 2) is achieved through the oxidation of the corresponding alkylthio substituted derivatives: 1,2-dicyano-4,5-bis (hexylsulfonyl) benzene **5** is prepared by a modified procedure^23^ from **4** with H_2_O_2_ in acetic acid at 79% yield. Iron(ii) octahexylsulfonyl phthalocyanine **6** is obtained by heating **5** in an *o*-dichlorobenzene : dimethylformamide (3 : 1) mixture commonly used for alkylsulfonyl phthalonitriles^24^ in the presence of FeCl_2_ (Scheme 2). The preparation of μ-nitrido diiron phthalocyanines is achieved by refluxing iron(ii) phthalocyanine in α-chloronaphthalene in the presence of NaN_3_.^25^ Such a reaction applied to an equimolar mixture of two differently substituted phthalocyanines results in a mixture of the two corresponding homoleptic complexes as well as the targeted heteroleptic derivative. To restrict the amount and yield of possible by-products, the heteroleptic μ-nitrido dimers **3** and **7** were prepared by using a 10-fold excess of unsubstituted iron phthalocyanine **1** relative to the monomeric alkylsulfonyl phthalocyaninatoiron(ii) **2** or **6**, respectively. These conditions allow the formation of the desired heteroleptic complexes (Scheme 2) together with the homoleptic unsubstituted derivative, which are separated by a procedure that starts with the evaporation of the α-chloronaphthalene, addition of dichloromethane, followed by filtration. Silica gel column chromatography afforded the desired heteroleptic complexes **3** and **7** in high yields (64 and 52% based on **2** and **6**, respectively). The lower yield of **7** with respect to **3** is attributed to a steric hindrance of the octasubstituted ligand **6**.

The successful preparation of **3** and **7** was confirmed by FT-IR, UV-vis and EPR spectroscopy and ESI-MS studies. The ESI-MS spectra of these mixed ligand complexes exhibit prominent molecular peaks corresponding to the expected values of the molecular ion: *m*/*z* = 1943.4 [M]^+^ for **3** and *m*/*z* = 2335.6 [M]^+^ for **7** (Fig. S1 and S5, ESI†). Their isotopic patterns fit the theoretical simulations perfectly and confirm the proposed structures. The IR spectra of **3** and **7** show characteristic anti-symmetric Fe–N–Fe stretch vibrations at 930 and 929 cm^–1^, respectively (Fig. S2 and S6, ESI†), which are close to those observed for the heteroleptic complex [(TPP)Fe^+3.5^NFe^+3.5^(Pc)] at 930 cm^–1^ and for homoleptic complex [(Pc)FeNFe(Pc)] at 915 cm^–1^.^14^−^18^

### X-ray absorption and emission spectroscopies

Over the past few years, synchrotron radiation core hole spectroscopy has shown great utility to address problems related to the oxidation state of iron in iron-containing enzymes and biomimetic iron structures.^26^ In previous work, we have successfully used X-ray absorption (XAS) and X-ray emission (XES) methods to gain insight into oxidation and spin states of μ-nitrido diiron phthalocyanines and compared electronic and structural properties of μ-carbido, μ-oxo and μ-nitrido diiron complexes.^27^ To probe a non-equivalence of iron sites in this work we performed detailed XES and XAS studies on heteroleptic complex **3** and homoleptic complex **8** as reference. Interestingly, the XES spectra of **3** and **8** are virtually identical in the solid phase (Fig. S9, ESI†), which implies that the two structures have the same metal coordination and oxidation state. Changes to the ligand system, therefore, appear to have had little effect on the electronic ground state of these diiron phthalocyanine systems.

### Extended X-ray absorption fine structure (EXAFS)

**Fig. 1:**
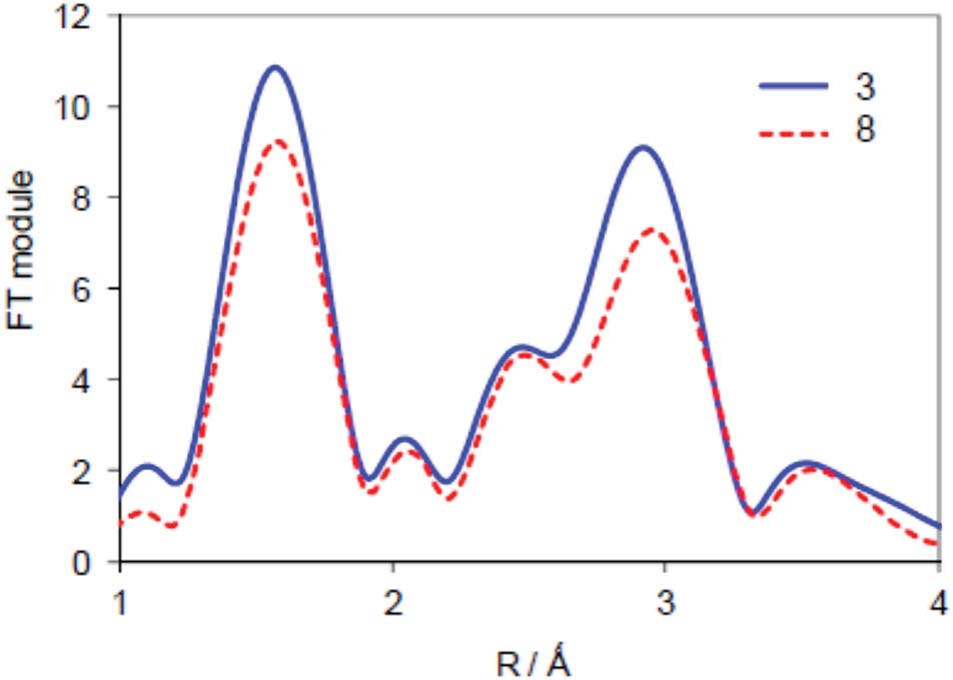
EXAFS spectra of the solid samples of heteroleptic complex **3** and homoleptic complex **8**.

In order to find out whether the different phthalocyanine rings lead to changes in the oxidation states of the iron atoms and the metal ligand distances, and in particular the bonds associated with the Fe–N–Fe moiety, we compared the Fe K-core hole spectra of complex **3** with the corresponding spectra of the symmetric structure **8**. The latter compound was extensively studied in our previous studies and proved to be a low-spin complex with equivalent iron centers as concerns both geometry and electronic orbital manifold.^15^ EXAFS spectra (Fig. 1) of **3** and **8** demonstrate striking similarity of iron coordination in the two complexes, which is characteristic of a linear Fe–N–Fe unit with Fe–N distances of 1.65 Å.

No significant differences in the interatomic distances of the heteroleptic complex **3** with respect to the homoleptic complex **8** can be inferred from the analysis of the EXAFS spectra (Fig. 1) in either *k*- or *R*-space. The only detectable difference between **3** and **8** is the somewhat larger intensity of the Fourier transfer (FT) module peaks of **3** for the first coordination shell. This is most likely due to differences in their Debye–Waller factors, as a result of more position isomers for **8** as compared to **3**. The effective disorder is therefore higher in **8**, and manifests itself in the EXAFS spectra with increased Debye–Waller parameters. Note that asymmetrization of the Fe–N–Fe fragment would lead to the decrease of Fe–N–Fe related features, as a result of weakening of the multiple scattering effects,^28^ whereas the opposite is observed here. Consequently, the EXAFS comparison of **3** and **8** reveals a symmetric Fe–N–Fe fragment in both complexes despite differences in the ligand properties in **3**.

### X-ray absorption near edge structure (XANES) studies

**Fig. 2:**
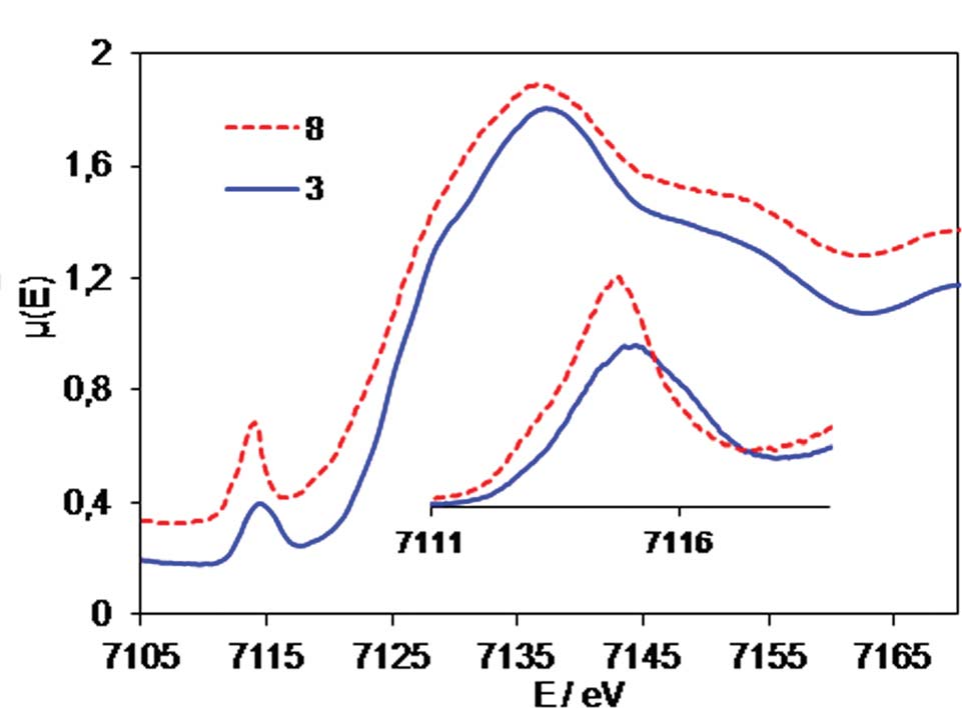
Experimental XANES spectra of heteroleptic complex **3** and homoleptic complex **8**. Inset – superposition of their pre-edge regions.

To gain further insight into the molecular and electronic properties of **3** and **8**, we performed a series of XANES experiments. In contrast to the XAS, XES and EXAFS studies reported above, the XANES studies reveal a difference between the two structures (Fig. 2). The energy values of the pre-edge peak in the XANES spectra of **3** and **8** are 7115.0 and 7114.7 eV, respectively. Most probably a 0.3 eV shift to higher energy for **3** arises due to the electron-withdrawing substituents on the phthalocyanine scaffold. The main jump for both compounds has a maximum derivative near 7126.1 eV. Due to complex structure of the main jump, the energies cannot be directly compared with the same precision as pre-edges.

### ESI-MS analysis of high-valent diiron–oxo complexes

**Scheme 3:**
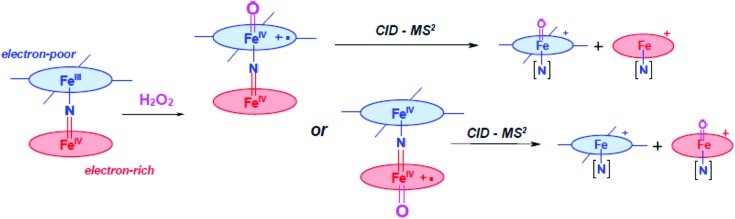
Formation of isomeric iron(iv)–oxo complexes with heteroleptic ligands and schematic representation of its fragmentation patterns. Blue ligand: substituted with **SO_2_R** groups. Red ligand: unsubstituted phthalocyanine.

Despite the initial high oxidation state of iron, the μ-nitrido diiron phthalocyanine complexes form the high-valent iron(iv)–oxo species readily in a reaction with active oxygen atom donors. The formation of the iron(iv)–oxo species, therefore, comprises of two fundamental steps: (i) coordination of the oxygen donor to the iron and (ii) heterolytic cleavage of the O–O bond of the peroxide unit. In the homoleptic complex both iron sites are identical and, as such, only one isomeric iron(iv)–oxo species can be formed. In the heteroleptic complex, by contrast, there are, in principle, two isomeric iron(iv)–oxo products possible (Scheme 3). The question arises then: can non-equivalence of the iron sites in terms of their electronic properties induce a discrimination in the formation of the iron(iv)–oxo complex? In order to find out whether a site-specific iron(iv)–oxo complex will be formed and what the properties of the ligands are that determine these issues, we performed a detailed combined ESI-MS and DFT study.

Highly sensitive mass spectrometry methods are particularly suitable for detection and characterization of elusive short lived active species.9e,^29^ In addition, advanced MS techniques allow one to obtain valuable mechanistic information on reaction mechanisms.^30^ In particular, tandem MS–MS method can be used to analyze a composition of diiron oxo complexes. To this aim, the molecular ion of the high-valent diiron–oxo species is mass selected and bombarded with neutral nitrogen gas. The collision induced dissociation of the parent ion leads to its fragmentation patterns into primary monomer units, which provides information on their composition, and enables structural characterization, and, in particular, will establish the iron site of the heteroleptic diiron complex that bears the oxo ligand. The optimal energy of the collision gas (N_2_) was determined to be ∼60 eV for the fragmentation of the diiron species into monomeric units without consecutive dissociation patterns. Fragmentation patterns of the heteroleptic compounds (Scheme 3) should give different products, depending on the nature of the phthalocyanine site coordinating the iron(iv)–oxo species, whether it is electron-rich or electron-deficient. Indeed, the analysis of the initial fragmentation pattern provides a composition of two different phthalocyanine species with or without oxo and/or nitrogen ligands, which enables us to conclude whether the oxo species is attached to the electron-deficient Fe(Pc**SO_2_R**) ligand or to the more electron-rich Fe(Pc) fragment (Scheme 3).

### Reaction of [(Pc**1**)FeNFe(Pc**2**)] (**3**) with oxidants

#### H_2_O_2_

**Fig. 3:**
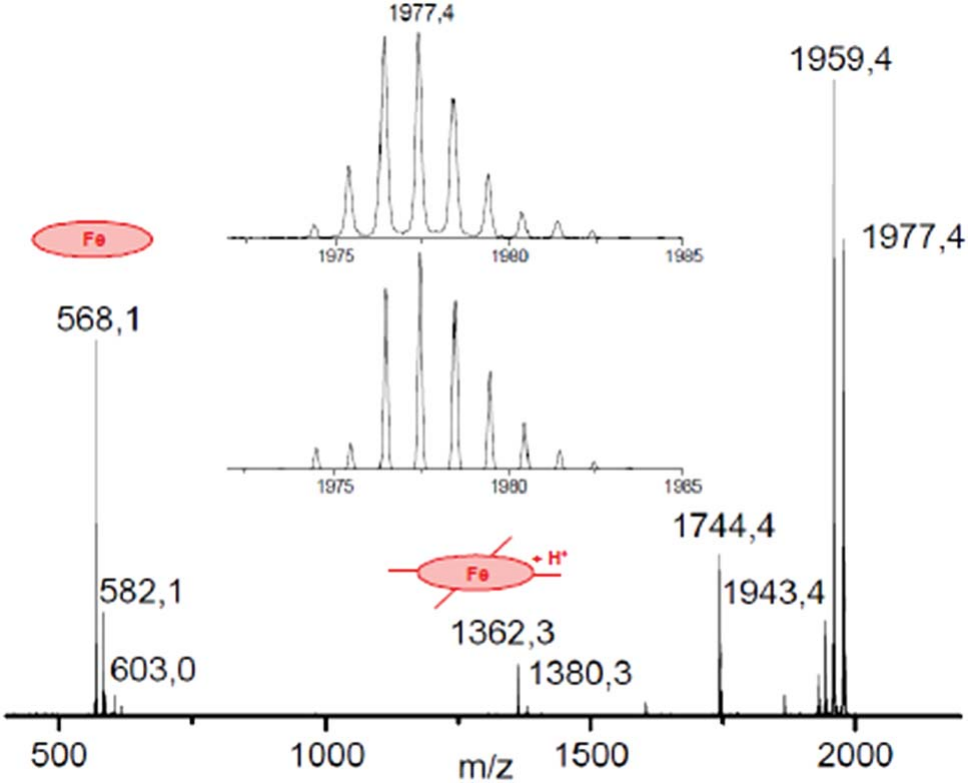
CID MS/MS spectrum of the hydroperoxo complex obtained from **3** and H_2_O_2_. Inset: comparison of experimental (top) and simulated (bottom) isotopic distribution of molecular peak.

**Fig. 4:**
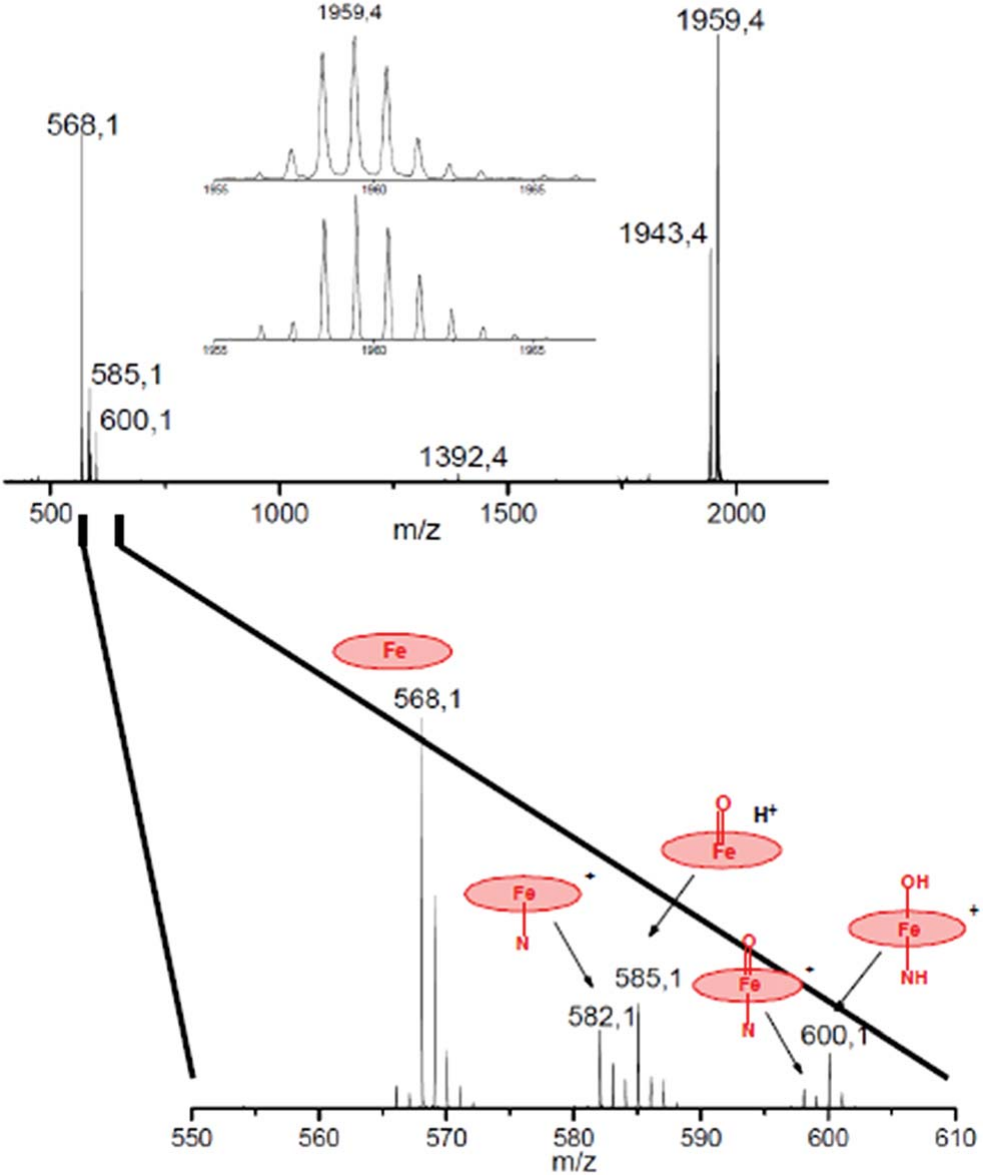
CID MS/MS spectrum of the oxo complex (**3=O**) obtained from **3** and H_2_O_2_. Inset: comparison of experimental (top) and simulated (bottom) isotopic distribution of molecular peak. An enlarged region of spectrum with assignment of fragments is shown below.

To a ∼10^–6^ M solution of **3** in CH_3_CN, 1000 equiv. of H_2_O_2_ was added at room temperature and the resulting solution was continuously introduced into the mass spectrometer. Along with a strong molecular peak of the initial complex **3** with *m*/*z* 1943.46, two signals centered at *m*/*z* 1977.46 and 1959.46 were observed that are assigned to [**3** + HOOH]^+^ and [**3** + O]^+^, respectively. The mass-selected peroxo and oxo species were further characterized by collision induced dissociation (CID MS/MS) and the results are schematically depicted in Fig. 3 and 4.

The principal ions formed upon fragmentation of [**3** + HOOH]^+^ (Fig. 3) are ions with mass representing [M – H_2_O]^+^ at *m*/*z* 1959.4, [M – H_2_O_2_]^+^ at *m*/*z* 1943.4 and [M – **SO_2_Ad**]^+^ at *m*/*z* 1744.4. In addition, fragments are observed containing monomeric phthalocyanine moieties. The abundance of fragments with the Fe(Pc**1**) core at *m*/*z* 582–585 and 603 containing N, OH and H_2_O_2_ ligands are much larger than those with Fe(Pc**2**) fragments that are found in the *m*/*z* range of 1376–1379 and 1397. The intensity of naked [Fe(Pc**1**)]^+^ ion at *m*/*z* 568 obtained after loss of oxygen ligands is also much higher than that of protonated [Fe(Pc**2**)+H]^+^ ion at *m*/*z* 1362.

The exact molecular mass and isotopic distribution pattern of [**3** + O]^+^ are identical to those theoretically predicted for the diiron–oxo complex (**3=O**), which confirms its formation. Upon CID MS/MS conditions, the **3=O** ion behaves very similarly to [**3** + HOOH]^+^ (compare Fig. 3 and 4). Again, dominant peaks in the *m*/*z* range of 568–600 are observed, which suggest site-selective oxygen binding to unsubstituted phthalocyanine unit. The molecular peak for [**3** + O]^+^ at *m*/*z* 1959.46 loses the oxygen ligand to give [(Pc**1**)FeNFe(Pc**2**)]^+^ (**3**) at *m*/*z* 1943.46. Importantly, the fragmentation of [**3** + O]^+^ produces almost exclusively [Fe(Pc**1**)]^+^ ions at *m*/*z* 568, 585, 600, whereas only minor amounts of [Fe(Pc**2**)]^+^ ions are detected at *m*/*z* 1362.

It should be noted here that fragments containing oxo ligands were only detected at the unsubstituted, *i.e.* more electron-rich, Fe(Pc**1**) platform. Signals centered at *m*/*z* 585 and 600 are attributed to [(Pc**1**)Fe=O + H]^+^ and [(Pc**1**)(N)Fe=O + 2H]^+^ ions on the basis of exact molecular mass and isotopic distribution of the cluster. A large signal at *m*/*z* 585 is attributed to either [(Pc**1**)(N)Fe=O + H^+^]^+^ or [(Pc**1**)(N)Fe–OH]^+^ ion formed by hydrogen atom abstraction from organic solvent by the oxo species with strong oxidizing properties. As expected, all these Fe(Pc**1**) oxygen containing species lose oxygen and nitrogen ligands to form naked [Fe(Pc**1**)]^+^ ions under CID-MS/MS conditions. The analysis of the fragmentation of [**3** + H_2_O_2_]^+^ and [**3** + O]^+^ ions suggests that high valent diiron species containing oxo ligands preferentially bind at more electron-rich iron sites such as Fe(Pc**1**).

#### *m*-CPBA

**Fig. 5:**
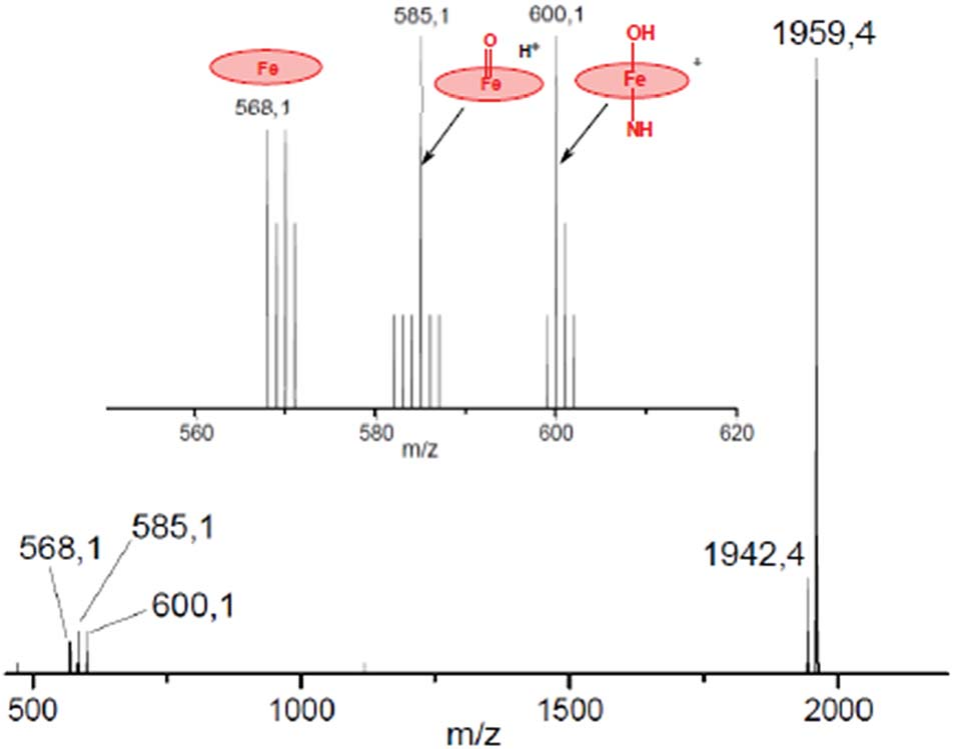
CID MS/MS spectrum of the oxo complex obtained from [(Pc**2**)FeNFe(Pc**1**)] and *m*-CPBA. An enlarged region of the spectrum with assignment of fragments is shown in the inset.

The diiron oxo complex **3=O** was also obtained using *m*-chloroperbenzoic acid as the oxygen donor. Importantly, the CID-MS/MS spectrum of **3=O**^*m*-CPBA^ shows only fragments derived from the Fe(Pc**1**) core (Fig. 5). The signals of Fe(Pc**1**) fragments bearing an oxo-ligand at *m*/*z* 585 and 600 are much stronger (Fig. 5) compared to those obtained from **3=O**^H_2_O_2_^ species (Fig. 4). This suggests that the oxo species most likely can be stabilized by acid *via* its coordination on the opposite iron site.^21^ We have also obtained hydroperoxo- and oxo diiron species of **7** using H_2_O_2_ and *m*-CPBA oxygen donors. Again, only Fe(Pc**1**) fragments with oxo-ligands are observed (ESI, Fig. S10 and S11†). Thus, all CID-MS/MS results obtained with two heteroleptic complexes **3** and **7** and with two oxidants H_2_O_2_ and *m*-CPBA indicate that the oxo complex is preferentially formed at the more electron-rich iron site of unsubstituted phthalocyanine ligand.

### Computational studies of **9**, [**9=O**] and [**9–OOH**]^–^

To support the experimental results and gain insight into the relative stability of μ-nitrido diiron phthalocyanines with different substitution patterns of the distal ligand, we performed a detailed density functional theory (DFT) study on structure **9**, which is an analogue of **3** whereby the adamantylsulfonyl groups are replaced by methylsulfonyl: [(Pc**1**)FeNFe(Pc**SO_2_Me**)]. In addition, we calculated the iron(iv)–oxo form of **9**, *i.e.* [**9=O**], and finally the iron(iii)–hydroperoxo complex [**9–OOH**]^–^. Although we calculated all structures in the lowest lying doublet, quartet and sextet spin states, actually in all cases the doublet spin state is the ground state and well separated from the other spin states. We, therefore, will focus in the main text on the doublet spin state only; all other results can be found in the ESI† document.

**Fig. 6:**
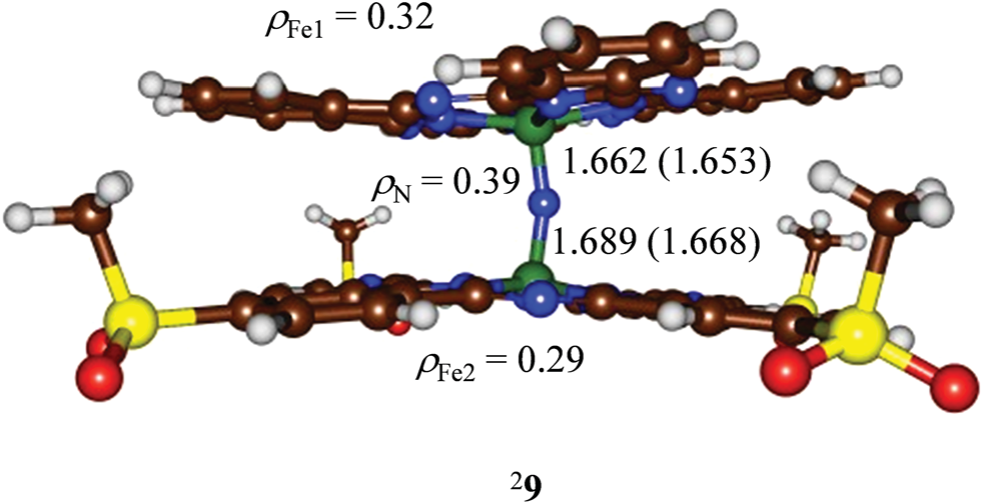
DFT optimized geometries of ^2^**9** at the B3LYP (BP86) level of theory. Bond lengths are in angstroms and spin densities (*ρ*) in atomic units.

We initially optimized the geometry of doublet spin **9** with UB3LYP and UBP86 methods and the structures (Fig. 6) show only small deviations from a symmetric Fe–N–Fe core with Fe–N distances of 1.689 (1.668) and 1.662 (1.653) Å at the B3LYP (BP86) levels of theory. These optimized geometries agree with the spectroscopic (EXAFS, XANES) results described above as well as with homoleptic structures calculated previously of 1.65 Å.27b,^31^ Although the optimized geometry implicates a structure that is close to symmetric, actually the minor differences between the two Fe–N bond lengths have a significant effect on the radical character of the two iron atoms. Thus, the iron of the Fe(Pc**1**) group has a spin of 0.32, whereas the other iron atom has a spin of only 0.29. This small change in radical character between the two iron atoms, due to the electron-withdrawing/donating groups attached to the phthalocyanine ligands may be responsible for the preferred site of hydrogen peroxide binding. In particular, the larger radical character of the Fe(Pc**1**) group will favour the binding of an active oxidant over the Fe(Pc**SO_2_Me**) group.

**Fig. 7:**
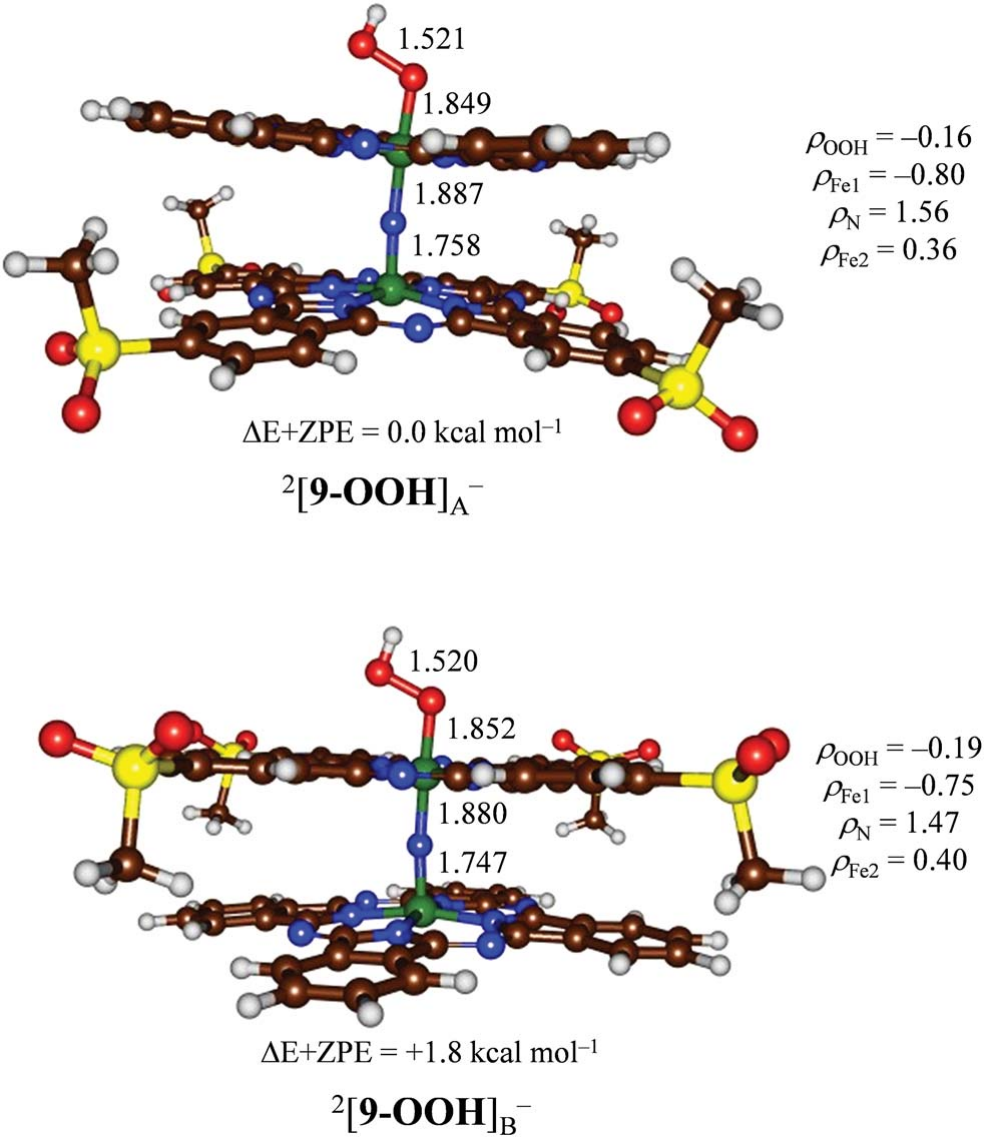
DFT optimized geometries of ^2^[**9–OOH**] at the B3LYP level of theory. Bond lengths are in angstroms and spin densities (*ρ*) in atomic units.

Subsequently, we added a hydroperoxo group to form [**9–OOH**]^–^ and reoptimized all geometries in the gas phase, see Fig. 7. We calculated two isomers, namely [HOO(Pc**1**)Fe^III^NFe^IV^(Pc**SO_2_Me**)]^–^, designated [**9–OOH**]_A_^–^, and [(Pc**1**)Fe^IV^NFe^III^(Pc**SO_2_Me**)OOH]^–^ or [**9–OOH**]_B_^–^. Similarly to previous studies on iron(iii)–hydroperoxo porphyrin complexes of mononuclear iron systems, the doublet spin state is the ground state in all cases.^32^ This doublet spin state is formed from the antiferromagnetic coupling of a triplet spin iron(iv)–nitrido group with an unpaired electron on the iron(iii)–hydroperoxo group. Although the energy gap between the two structures is small, the most stable isomer is structure [**9–OOH**]_A_^–^ by 1.8 kcal mol^–1^ over [**9–OOH**]_B_^–^. Therefore, the iron(iii)–hydroperoxo is preferentially bound to the site with electron-donating substituents attached to the phthalocyanine manifold. This is a surprising result as previous computational and experimental studies found very little effect, for instance, of meso-substitution of porphyrin rings on the electron affinity of the iron(iv)–oxo group.^33^ Nevertheless, the stability difference of [**9–OOH**]_A_^–^*versus* [**9–OOH**]_B_^–^ confirms the experimental MS results described above that more fragmentation is found from the [**9–OOH**]_A_^–^ isomer.

The group spin densities reported in Fig. 7 for [**9–OOH**]_A_^–^ and [**9–OOH**]_B_^–^ show some critical differences between the two structures. Thus, the iron(iii)–hydroperoxo group has larger radical character on the metal when it has a ligand with electron-donating substituents (Pc**1**) than one with electron-withdrawing substituents (Pc**SO_2_Me**): *ρ*_Fe_**_1_** = –0.80 for [**9–OOH**]_A_^–^ and *ρ*_Fe_**_1_** = –0.75 for [**9–OOH**]_B_^–^. The same is true for the iron(iv)–nitrido group that sees the spin density polarize more towards the iron with the Pc**1** ligand rather than the Pc**SO_2_Me** ligand. Therefore, electron-withdrawing substituents on the phthalocyanine scaffold create metal centers with lesser radical character and as such should be less reactive.

In summary, the computational results implicate preferential binding on the iron center with electron-donating substituents by a few kcal mol^–1^, however, it is not ruled out that a mixture of isomers will be formed in the process dependent on temperature, pressure, solvent environment *etc.* Indeed, the mass spectra show the presence of both type **A** and type **B** complexes as major and minor species, respectively.

High-valent iron complexes bearing iron–oxygen^5^−^9^ or iron–nitrogen^34^ multiple bonds have been well-documented in the literature as active species involved in a variety of oxidative transformations. In contrast, high-valent iron complexes containing both oxo and nitrido/imido ligands are rare entities. μ-Nitrido diiron macrocyclic complexes are particularly interesting since they combine this rare structural feature, which results in remarkable catalytic properties.^13^,^14^,^21^ These μ-nitrido diiron macrocyclic complexes generally contain equivalent iron sites despite the fact that both metals formally have a different oxidation state, *i.e.* Fe^III^Fe^IV^.^18^ On the other hand, studies on related μ-oxo/μ-hydroxo diiron porphyrin complexes show that they often have non-equivalent iron sites, whereby their oxidation states are very sensitive to structural and environmental perturbations.^12^,^35^ This delocalized structure retains even in heteroleptic porphyrin–phthalocyanine dimers.22*b* To probe the non-equivalence of iron sites in the heteroleptic μ-nitrido diiron phthalocyanine complexes we applied XAS and XES techniques. The XES spectra of the heteroleptic **3** and reference homoleptic **8** complexes are practically the same, which indicates little or no effect of desymmetrization of the diiron unit. However, the CID-MS/MS data on [**3=O**] clearly indicate the preferential formation of an iron(iv)–oxo species at the more electron-rich, *i.e.* unsubstituted, phthalocyanine unit (Pc**1**). Noteworthy, the same trend was observed with either heteroleptic dimer **3** or **7** and too was found to be independent on the nature of the oxygen donor, *i.e.* H_2_O_2_ or *m*-CPBA.

To find out, whether there are stability differences for μ-nitrido diiron–oxo complexes with heteroleptic ligand systems as well, we decided to do an additional DFT study into the two [**9=O**] isomers, namely [O=Fe^IV^(Pc**1**)NFe^IV^(Pc**SO_2_Me**)] or [**9=O**]_A_ and [Fe^IV^(Pc**1**)NFe^IV^(Pc**SO_2_Me**)=O] or [**9=O**]_B_. The structures of [**9=O**]_A_ and [**9=O**]_B_ were calculated with B3LYP and BP86. The B3LYP and BP86 optimized structures show dramatic differences in chemical structure but follow the same trends upon adding substituents to one of the phthalocyanine scaffolds. Firstly, the iron–oxo bond is short with B3LYP (1.647 Å for structure [**9=O**]_A_ and 1.652 Å for structure [**9=O**]_B_), which implicates an iron(iv)–oxo double bond. These values match computational and experimental measurements of mononuclear iron(iv)–oxo complexes of porphyrin systems excellently.^5^−^9^,^36^ Furthermore, the iron(iv)–oxo bond length drops by 0.005/0.004 Å at B3LYP/BP86 level of theory when electron-withdrawing substituents are added at a distance of more than 8 Å from the iron center in the equatorial plane. Consequently, a significant difference in iron–oxo bond length is obtained between structures [**9=O**]_A_ and [**9=O**]_B_, which leads to stability differences and may be the primary reason for the difference in stability between doublet [**9=O**]_A_ and [**9=O**]_B_ structures. A change of this magnitude was not observed previously in meso-substituted iron porphyrins.^33^ Therefore, phthalocyanines are more sensitive to substitution patterns on the periphery of the ligand system and this has a direct effect on a generated iron(iv)–oxo species.

**Fig. 8:**
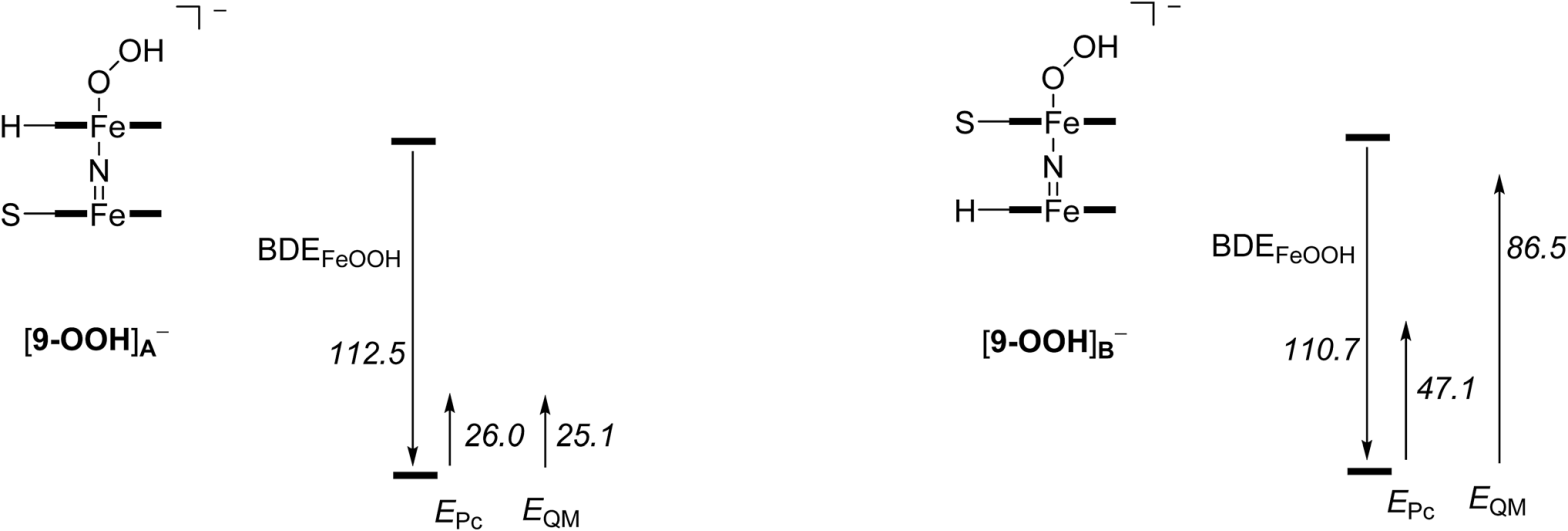
Effect of the axial ligand and second phthalocyanine group on the binding strength of the hydroperoxo moiety. All values are in kcal mol^–1^.

To establish the origin of the hydroperoxo binding to [(Pc**1**)FeNFe(Pc**SO_2_Me**)], we decided to calculate the bond dissociation energy (BDE) of the hydroperoxo unit in [**9–OOH**]_A_^–^ and [**9–OOH**]_B_^–^ based on eqn (1). The calculated values for both complexes are given in Fig. 8, where we bisected the BDE_FeOOH_ into different contributions as explained below. Of course, the BDE_FeOOH_ difference between [**9–OOH**]_A_^–^ and [**9–OOH**]_B_^–^ is the same as the stability difference reported in Fig. 7.

1 [**9–OOH**]^–^ → **9** + OOH^–^ + BDE_FeOOH_

Subsequently, we removed the phthalocyanine group of the N=Fe^IV^(Pc) moiety and replaced it by (NH_3_)_2_(NH_2_^–^)_2_ with these nitrogen atoms in the same position as the nitrogen atoms of the phthalocyanine group. A single point calculation for these modified structures for **9**, [**9–OOH**]_A_^–^ and [**9–OOH**]_B_^–^ gives the effect of the second Pc unit on the BDE values for eqn (1), *E*_Pc_, namely through π–π stacking interactions. As can be seen, the size and shape of the second Pc group has a major impact on the BDE_FeOOH_ value, even though this bond is at a distance of well over 4 Å. Thus, the BDE_FeOOH_ is strongly increased for the (Pc**SO_2_Me**)FeOOH system as compared to the (Pc**H**)FeOOH system by more than 20 kcal mol^–1^.

In a final set of calculations, we removed the N=Fe^IV^(Pc) unit altogether and replaced it by a point charge. Again a single point calculation was performed to determine the relative BDE_FeOOH_ values using eqn (1). These values establish the quantum mechanical effect (*E*_QM_) of the axial ligand upon the Fe–OOH binding energy. Thus, the axial N=Fe^IV^(Pc**SO_2_Me**) group gives a quantum mechanical effect that is dominated by the presence of the Pc**SO_2_Me** interaction with the rest of the system and has little contribution from the Fe^IV^=N moiety. By contrast, the removal of the N=Fe^IV^(Pc**H**) group has a major effect on the BDE_FeOOH_ value and the system is considerably stabilized. Clearly, the equatorial ligands have a major contribution towards the binding of hydroperoxo groups to an iron scaffold and strengthen its bonding. This further shows that electron-withdrawing and electron-donating substituents can be used to manipulate the binding site of (hydro)peroxo moieties, and affect their stability and ultimately their catalytic properties.

### Comparison of μ-nitrido diiron complexes in the oxidation of methane by H_2_O_2_

To check the conclusions of ESI-MS and computational studies on the influence of the structure of μ-nitrido diiron species on the catalytic properties, we have tested homoleptic complexes [FePc(SO_2_^*t*^Bu)_4_]_2_N, (FePc)_2_N and [FePc(^*t*^Bu)_4_]_2_N (**8**) in the oxidation of methane. The complexes were supported onto silica (loading = 20 μmol g^–1^, specific surface = 200 m^2^ g^–1^) and the heterogeneous oxidation of methane was performed in water containing 75 mM H_2_SO_4_ at 60 °C (Table 1).

**Table 1:** Heterogeneous oxidation of methane by H_2_O_2_ catalyzed by μ-nitrido diiron phthalocyanine complexes^*a*^

Entry	Catalyst	TON^*b*^	HCOOH, μmol	CH_2_O, μmol
1	[FePc(^*t*^Bu)_4_]_2_N–SiO_2_	223.4	216.0	7.4
2	(FePc)_2_N–SiO_2_	102.9	90.0	12.9
3	[FePc(SO_2_^*t*^Bu)_4_]_2_N–SiO_2_	32.1	28.6	3.5

^*a*^Reaction conditions: 2 mL of 75 mM H_2_SO_4_, 50 mg of catalyst containing 1 μmol of the complex, 32 bar methane, 685 μmol H_2_O_2_, 60 °C, 20 h.

^*b*^TON – turnover number, μmol of products/μmol of catalyst.

With all the catalysts, methane was efficiently oxidized to formic acid with 88–97% selectivity. Methanol was detected by GC-MS in trace amounts and formaldehyde is readily oxidized under the reaction conditions. Interestingly, with growth of the electron-donating properties of phthalocyanine ligand along the series [FePc(SO_2_^*t*^Bu)_4_]_2_N < (FePc)_2_N < [FePc(^*t*^Bu)_4_]_2_N the values of total turnover numbers (μmol of products/μmol of catalyst) strongly increase: 32.1 → 102.9 → 223.4. These turnover numbers reflect the difference in O–H bond strength of iron(iii)–hydroxo complexes with different phthalocyanine groups. Thus, previously we showed that the rate constant of hydrogen atom abstraction reactions correlates linearly with the O–H bond dissociation energy of the corresponding iron(iii)–hydroxo complex formed.^37^ In agreement with the ESI-MS and DFT results, the μ-nitrido dimer on the unsubstituted phthalocyanine platform was 3 times more active than the complex with alkylsulfonyl substituents. Moreover, further increase of the electron density at Fe sites led to significant gain in the catalytic activity. The [FePc(^*t*^Bu)_4_]_2_N complex bearing donating alkyl substituents showed an excellent performance in the mild methane oxidation providing a very high turnover number of 223.

## Conclusion

In addition to remarkable catalytic properties exemplified by mild oxidation of methane and unprecedented activation of the aromatic C–F bonds under oxidative conditions,^14^ diiron macrocyclic platforms provides novel, previously unexplored possibilities to examine fine mechanistic details. In this work, two diiron phthalocyanine complexes with electronically different iron sites have been synthesized and used for the preparation of high-valent diiron oxo active species possessing powerful oxidizing ability. Such a construction provides direct intra-molecular approach to assess the reactivity of iron sites in binuclear complexes. Combined EXAFS, XANES, ESI-MS and DFT studies show that the hydroperoxo oxidant will preferentially bind on the more electron-rich iron site, which will then also be the place where the oxo species is formed. The results of this study are instructive for the development of μ-nitrido diiron catalysts. Since the formation of diiron oxo species occurs at the electron-rich site of the dimer, complexes bearing electron-donating groups might provide better catalytic properties. This prediction was confirmed in the catalytic oxidation of methane by H_2_O_2_ in aqueous solution using μ-nitrido diiron complexes supported by different phthalocyanine ligands. Indeed, the catalyst with electron-donating alkyl substituents provided a remarkably high TON of 223 compared with TON of 32 obtained using the counterpart with electron-withdrawing properties. Based on the results of this study, the further development of the bio-inspired catalysts with improved catalytic properties for challenging reactions can be envisaged.

## Experimental

### General methods

Infrared spectra (IR) were recorded on a Bio-Rad FTS 175C FTIR spectrophotometer. UV-visible absorption spectra were obtained using a Shimadzu 2001 UV spectrophotometer. High resolution mass spectra were measured on a Bruker microTOF spectrometer equipped with electrospray ionization (ESI) source. ^1^H spectra were recorded in deuterated chloroform (CDCl_3_) on a Varian 500 MHz spectrometer. X-band (9.5 GHz) EPR spectra were recorded at 77 K on a Bruker ESP 300E spectrometer using a standard rectangular (TE102) EPR cavity (Bruker ER4102ST) with a microwave power of 1.6 mW and modulation amplitude of 1 G.

The mass spectra were recorded on a MicroTOF Q II (Bruker Daltonics, Bremen), a quadrupole time of flight (Q-TOF) mass spectrometer, equipped with a cold spray ionization source with a spray voltage of 5 kV. The temperature of the spray gas was –30 °C and that of the dry gas was –5 °C. The solutions of the complexes (10^–6^ M) in acetonitrile in the presence of 1000 equiv. of oxidant were introduced into the gas flow at 180 μL h^–1^. In experiment with H_2_O_2_ oxidant, the reaction mixtures were prepared and incubated for 5 min at room temperature. The *m*-CPBA oxidant was added to the frozen complex solution in acetonitrile and the sample was introduced immediately after melting. All mass spectra were recorded in the positive ion mode.

The molecular ions (M^+^) of the oxo and peroxo diiron phthalocyanine species observed in the regular MS experiment cannot be trapped in the quadrupole region due to the formation of multimers by π-stacking interactions. To overcome this problem, a low activation of ions during transmission through the ion funnels was applied by In Source Collision Induced Dissociation (ISCID) at 20 eV, which enabled us to do MS/MS measurements. This activation allowed us to mass select the oxo and peroxo diiron phthalocyanine species in the quadrupole part of the mass spectrometer. The collision energy applied in the collision cell was about 60 eV and the collision gas was nitrogen.

### Materials

Solvents were dried and distilled according to published procedures.^38^ Hydrogen peroxide (35%) was obtained from Sigma-Aldrich. *m*-Chloroperbenzoic acid (*m*-CPBA) purchased from Sigma-Aldrich with 85% peroxide content was recrystallized from pentane prior to use. Phthalocyaninatoiron(ii) (**1**),^39^ tetra-(adamantylsulfonyl) phthalocyaninatoiron(ii) (**2**),^40^ 1,2-dicyano-4,5-bis (hexylthio) benzene (**4**),^41^ homoleptic complex **8**,14*a* (FePc)_2_N,^25^ and [FePc(SO_2_^*t*^Bu)_4_]_2_N,^16^ were prepared according to literature protocols.

#### 1,2-Dicyano-4,5-bis(*n*-hexylsulfonyl)benzene (**5**)

1,2-Dicyano-4,5-bis(hexylthio)benzene (**4**) (1.5 g, 4.16 mmol) was stirred in acetic acid (50 mL) at 90 °C, and subsequently a 33% solution of hydrogen peroxide (23 mL) was added in 1 mL portions over 4 h. The resulting mixture was allowed to cool to room temperature and stirred overnight. The resulting white precipitate was collected by filtration, washed with water and crystallized from ethanol (30 mL) to give **5** as a white powder. Yield 79% (1.4 g). Mp 113–114 °C. Anal. calcd for C_20_H_28_N_2_O_4_S_2_: C, 66.62; H, 7.83; N, 7.77; S, 17.79. Found: C, 67.20; H, 7.70; N, 7.93; S, 17.57. ^1^H NMR (500 MHz CDCl_3_): *δ* = 0.8 (t, 3H, CH_3_), 1.3–1.7 (m, 8H, CH_2_), 3.7 (t, 2H, CH_2_), 8.6 (s, 1H, ArH) ppm. ESI-MS: *m*/*z*: 447.23 [M + Na]^+^; IR (KBr) *ν* (cm^–1^): 3097, 2959, 2928, 2861, 2244, 1545, 1464, 1336, 1314, 1149, 1125, 919, 795, 728, 648, 570, 525, 512, 474.

#### Octa-(*n*-hexylsulfonyl)phthalocyaninato iron(ii) (**6**)

A mixture of 1,2-dicyano-4,5-bis(*n*-hexylsulfonyl)benzene (**5**) (250 mg, 0.6 mmol) was heated at 130 °C in a mixture of *o*-dichlorobenzene–DMF (3 : 1) under argon for 8 h in the presence of iron(ii) chloride (0.15 mmol). The solvent was then concentrated under reduced pressure and the resulting green solid was recovered with dichloromethane and washed with water. Phthalocyanine **6** was purified by chromatography on silica gel using a mixture of di-chloromethane : ethanol (100 : 1) as the eluent. Yield: 23% (61 mg). ESI-MS: *m*/*z* calc. for C_80_H_112_FeN_8_O_16_S_8_, 1172.5; found 1753.3 [M + H]^+^. UV-vis (CHCl_3_): *λ*_max_ (log *ε*) = 670 (5.2), 354 (5.0) nm; IR (KBr) *ν* (cm^–1^): 1310, 1100, 1070, 808, 740.

#### General procedure for preparation of heteroleptic N-bridged diiron phthalocyanine dimers

FePc**1** (1 mmol), sodium azide (1.5 g) and alkylsulfonyl phthalocy-aninato-iron(ii) (0.1 mmol) were suspended in α-chloronaphthalene (20 mL) under argon. The mixture was heated for 24 h at 190 °C under intensive stirring. The solvent was removed under reduced pressure. The remaining blue solid was diluted in CH_2_Cl_2_ (100 mL), filtrated and concentrated to be loaded on a silica gel column first eluted with CH_2_Cl_2_ to remove impurities. Then, the desired heteroleptic dimer was collected using CH_2_Cl_2_ : EtOH (10 : 1) as the eluent. Evaporation of solvent afforded the pure heteroleptic dimer (**3** or **7**, see below) as a dark blue powder.

#### [(Pc1)FeNFe(Pc2)] (**3**)

Yield: 64% (124 mg). ESI-HRMS: *m*/*z* calc. for C_104_H_88_Fe_2_N_17_O_16_S_8_, 1943.8; found, [M]^+^, dicharged species calc. for 971.2287; found 971.2332. UV-vis (CHCl_3_): *λ*_max_ (log *ε*) = 635 (4.5), 338 (4.3) nm. IR (KBr) *ν* (cm^–1^): 929 (Fe=N–Fe).

#### [(Pc1)FeNFe(Pc6)] (**7**)

Yield: 52% (120 mg). ESI-HRMS: *m*/*z* calc. for C_112_H_128_Fe_2_N_17_O_16_S_8_, 2334.6186; found, 2334.6091 [M]^+^. UV-vis (CHCl_3_): *λ*_max_ (log *ε*) = 645 (4.3), 338 (4.1) nm. IR (KBr) *ν* (cm^–1^): 935 (Fe=N–Fe).

### Catalytic oxidation of methane

The [FePc(SO_2_^*t*^Bu)_4_]_2_N, (FePc)_2_N and [FePc(^*t*^Bu)_4_]_2_N complexes were supported onto silica (Degussa aerogel, 200 m^2^ g^–1^) with loading of 20 μmol g^–1^ according to a published procedure.14a,b The heterogeneous oxidation of methane was performed as previously described.14a,b

### Computational methods

Density functional theory (DFT) procedures were employed using previously calibrated and benchmarked methods^42^ as implemented in the Gaussian-09 and Jaguar software packages.^43^ Initially, structures were constructed *ab initio* and optimized using the unrestricted BP86 and B3LYP density functional methods^44^,^45^ and basis set BS1,^46^ which consists of LANL2DZ on Fe and 6-31G on the other atoms. We set up an abbreviated model of structure **3**, namely structure **9** [(Pc**H**)Fe^III^NFe^III^(Pc**SO_2_Me**)], whereby the adamantyl-sulfonyl groups were replaced by SO_2_CH_3_ as shown in Scheme 1. In addition, structures were calculated with one iron(iv)–oxo group, namely [(Pc**H**)Fe^III^NFe^IV^(Pc**SO_2_Me**)=O] (**9=O**_A_) and [O=(Pc**H**)Fe^IV^NFe^III^(Pc**SO_2_Me**)] (**9=O**_B_). Furthermore, structures were generates with an iron(iii)–hydroperoxo group, *i.e.* [(Pc**H**)Fe^III^NFe^III^(Pc**SO_2_Me**)OOH] (**9–OOH**_A_) and [HOO(Pc**H**)Fe^III^NFe^III^(Pc**SO_2_Me**)] (**9–OOH**_B_). All individual structures were calculated in the lowest energy doublet, quartet, and sextet spin states, but the doublet spin state is the ground state in all cases.

To test the effect of the basis set on the spin state ordering and relative energies, we calculated single points with a triple-*ζ* type basis set on iron (LACV3P+) and 6-311+G* on the rest of the atoms: basis set BS2. Finally, solvent corrections were obtained from single point calculations in Jaguar using the polarized continuum model with a dielectric constant mimicking dichlorobenzene. However, none of these calculations changed the spin state ordering or site preference, see ESI† for details.

These DFT methods have been extensively used in calculations of iron–porphyrin complexes and were shown to reproduce experimental rate constants and spin state orderings in good agreement with experiment.^47^ Each chemical structure was characterized as a local minimum *via* a harmonic frequency calculation, which gave real vibrational frequencies only.

## Supplementary Material

Supplementary information
