# Supplementary material for: Site-selective formation of an iron(iv)–oxo species at the more electron-rich iron atom of heteroleptic μ-nitrido diiron phthalocyanines
Source: Chem Sci. 2015 Jun 16;6(8):5063–75. doi: 10.1039/c5sc01811k (PMC6088558; doi:10.1039/c5sc01811k)
Supplement: Supplementary file 1 [file SC-006-C5SC01811K-s001.pdf]

Electronic Supplementary Material (ESI) for Chemical Science.
This journal is © The Royal Society of Chemistry 2015

Electronic Supporting Online material for

Site-selective formation of an iron(IV)-oxo species at the more electron-rich iron atom of heteroleptic μ-nitrido diiron Phthalocyanines.

Ümit İşci,[a] Abayomi S. Faponle,[b] Pavel Afanasiev,[c] Florian Albrieux,[d] Valérie Briois,[e] Vefa Ahsen,[a] Fabienne Dumoulin,*[a] Alexander B. Sorokin,*[c] and Sam P. de Visser*[b]

alexander. sorokin@ircelyon.univ-lyon1.fr, sam.devisser@manchester.ac.uk,
fdumoulin@gtu.edu.tr

This PDF file includes:

Supplementary Figures S1 to S12

Tables, Figures and Cartesian coordinates with Computational data.

[a.] Gebze Technical University, Department of Chemistry, P.O. Box 141, Gebze, 41400 Kocaeli, Turkey.
[b.] Manchester Institute of Biotechnology and School of Chemical Engineering and Analytical Science, The University of Manchester, 131 Princess Street, Manchester M1 7DN, United Kingdom.
[c.] Institut de Recherches sur la Catalyse et l'Environnement de Lyon (IRCELYON), UMR 5256, CNRS-Université Lyon 1, 2, av. A. Einstein, 69626 Villeurbanne Cedex, France.
[d.] Centre Commun de Spectrométrie de Masse UMR 5246, CNRS-Université Claude Bernard Lyon 1, Université de Lyon, Bâtiment Curien, 43, bd du 11 Novembre, 69622 Villeurbanne Cedex, France.
[e.] Synchrotron Soleil, L'orme des merisiers, St-Aubin, 91192 Gif-sur-Yvette, France.
† Footnotes relating to the title and/or authors should appear here.
Electronic Supplementary Information (ESI) available: Experimental and computational details, Cartesian coordinates, characterization data, and ESI-MS spectra. See DOI: 10.1039/x0xx00000x

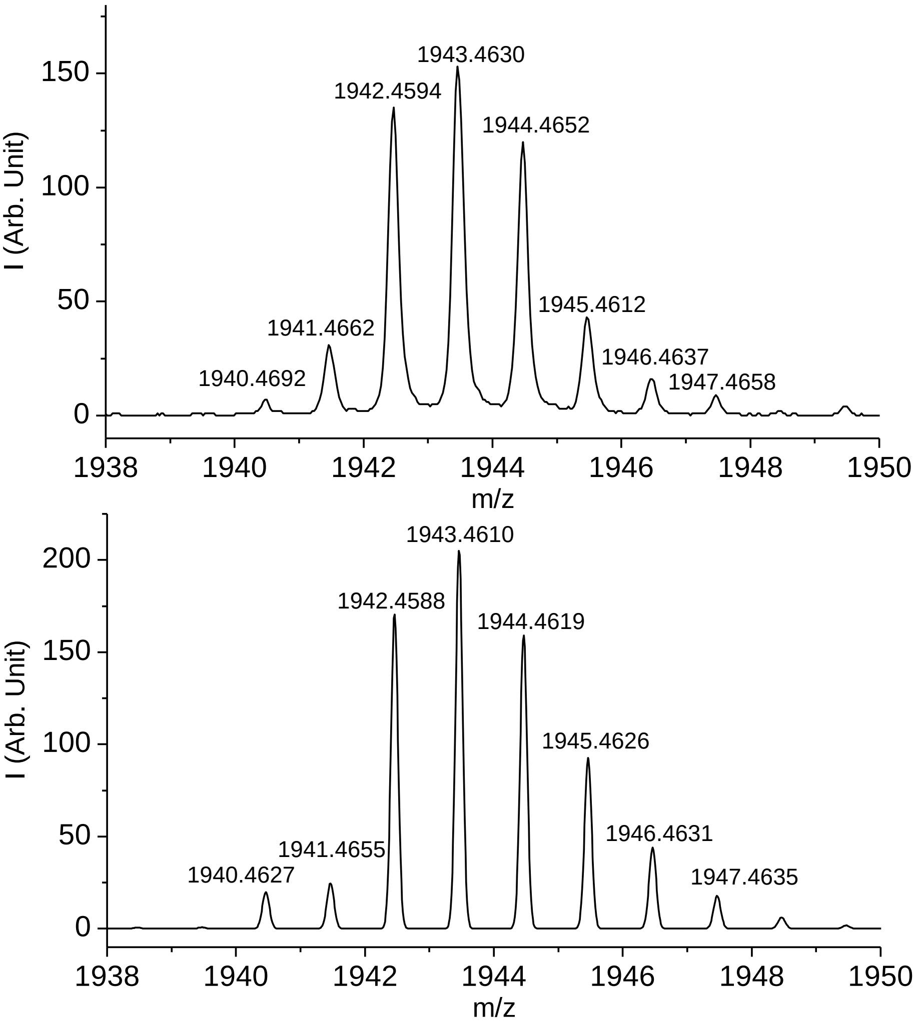

Figure S1. High resolution ESI-MS characterisation: experimental and theoretical molecular peak clusters of **3** observed as doubly-charged ions.

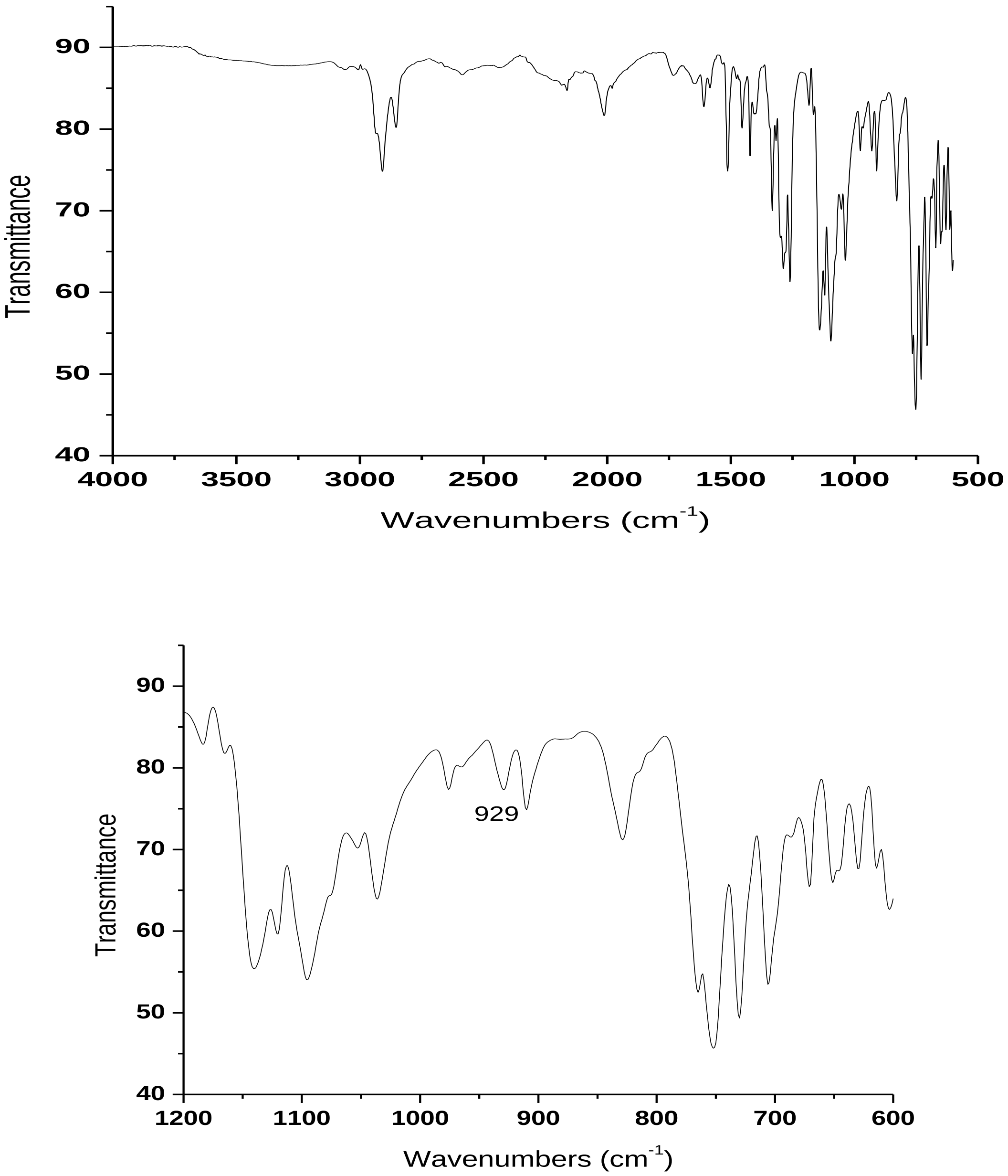

Figure S2. Top: full FT-IR spectrum of **3**. Bottom: zoom in at the 1200 – 600 cm^{-1} region.

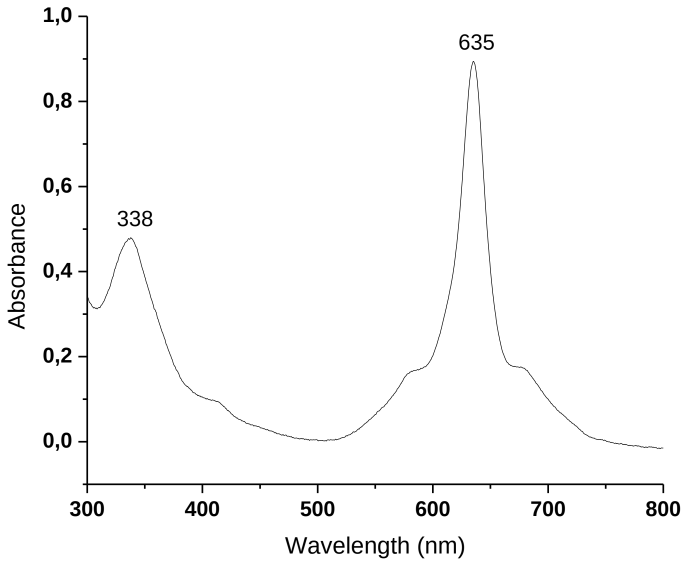

Figure S3. UV-Vis spectrum of **3** in $CHCl_3$ (10 μM).

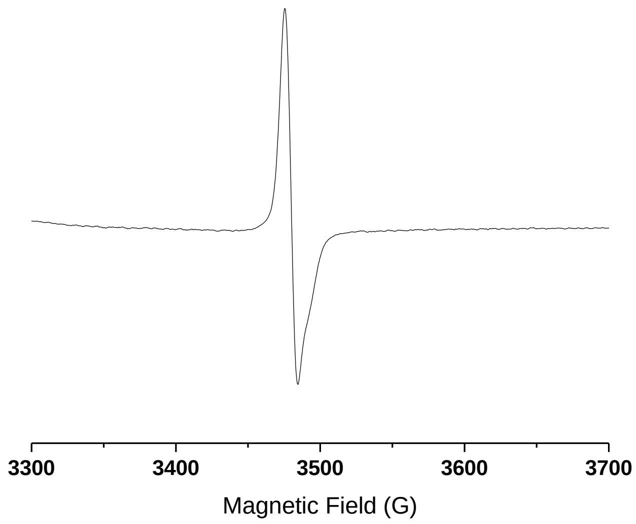

Figure S4. X-Band 77 K EPR spectrum of **3** (g = 2.05).

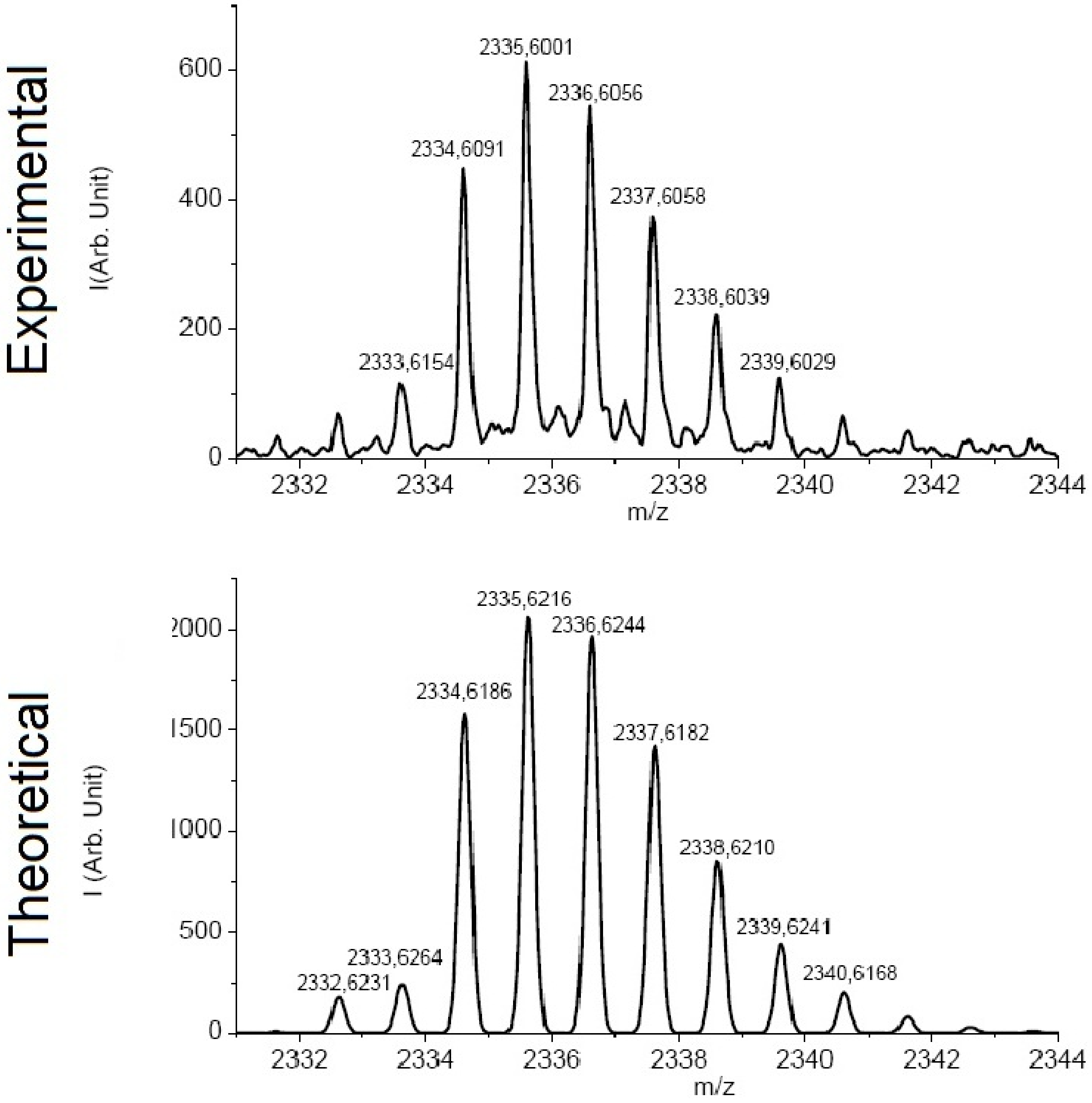

Figure S5. High resolution ESI-MS characterisation: experimental and theoretical molecular peak clusters of **7**.

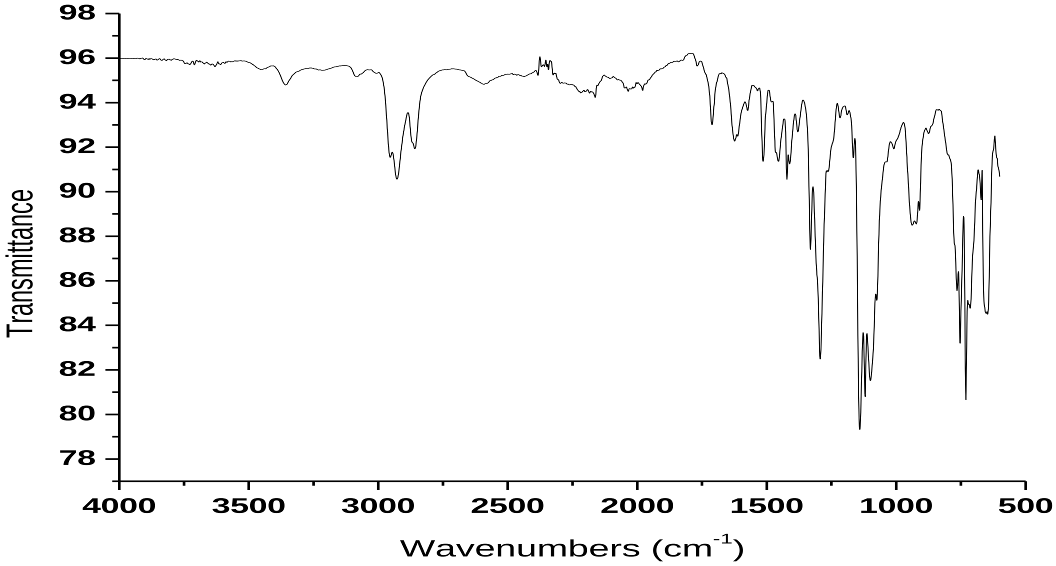

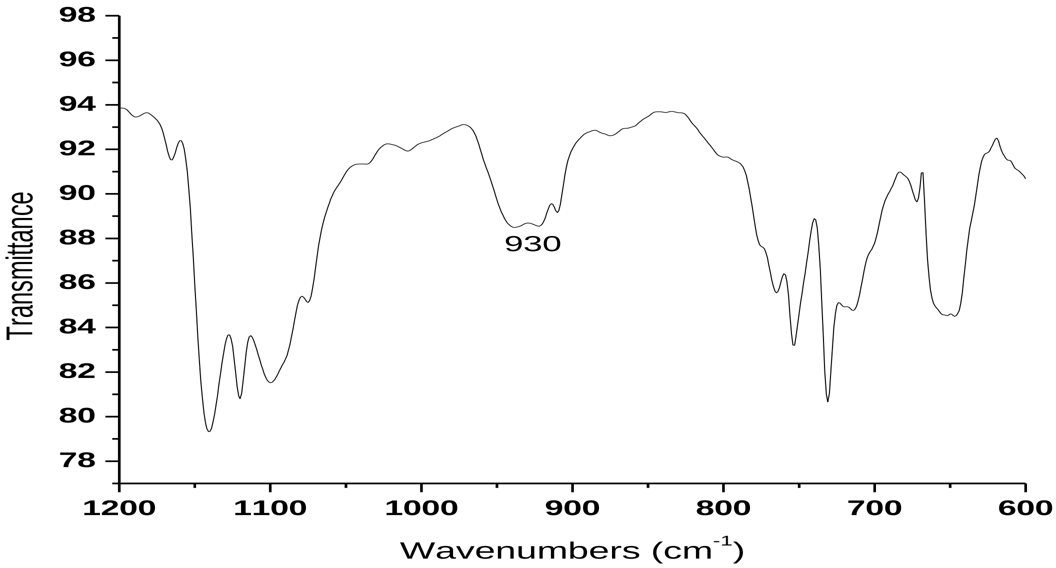

Figure S6. Top: full FT-IR spectrum of **7**. Bottom: zoom in at the 1200 – 600 cm^{-1} region.

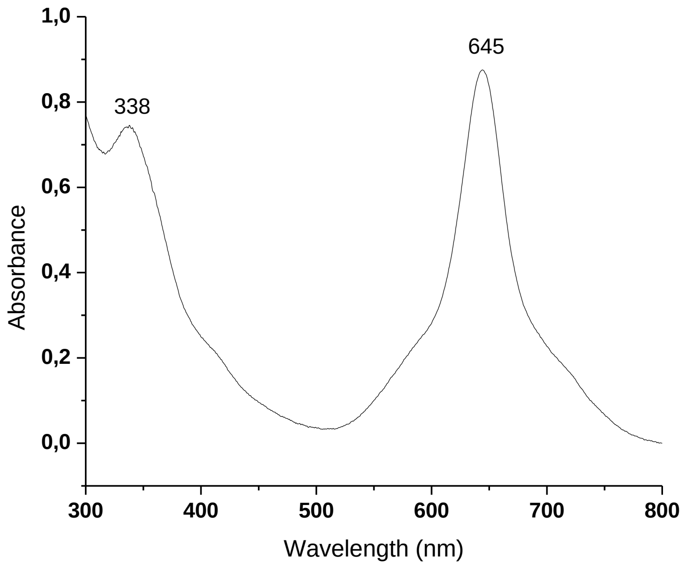

Figure S7. UV-Vis spectrum of **7** in $CHCl_3$ (10 μM).

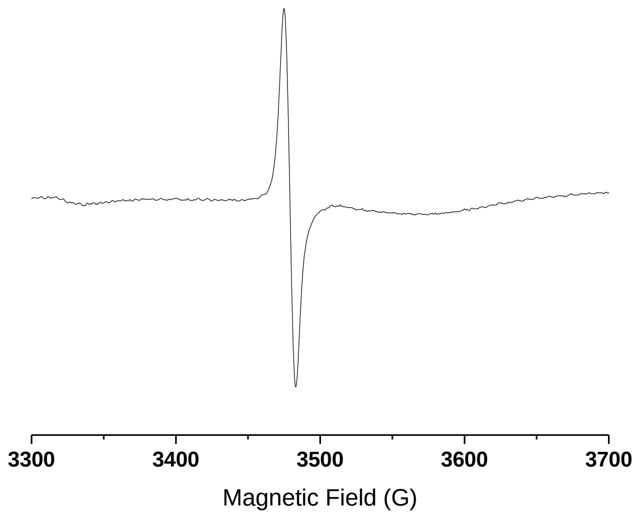

Figure S8. X-Band 77 K EPR spectrum of **7** (g = 2.05).

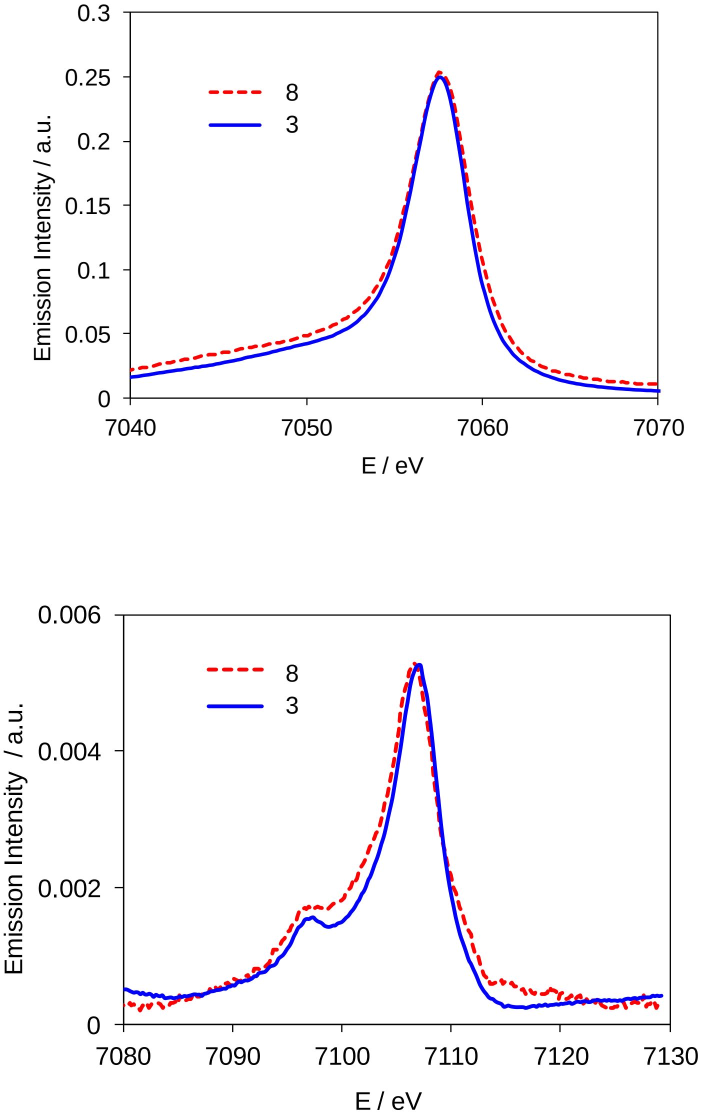

Figure S9. High resolution Kβ emission spectra in the main and satellite regions for the complexes **3** and **8**.

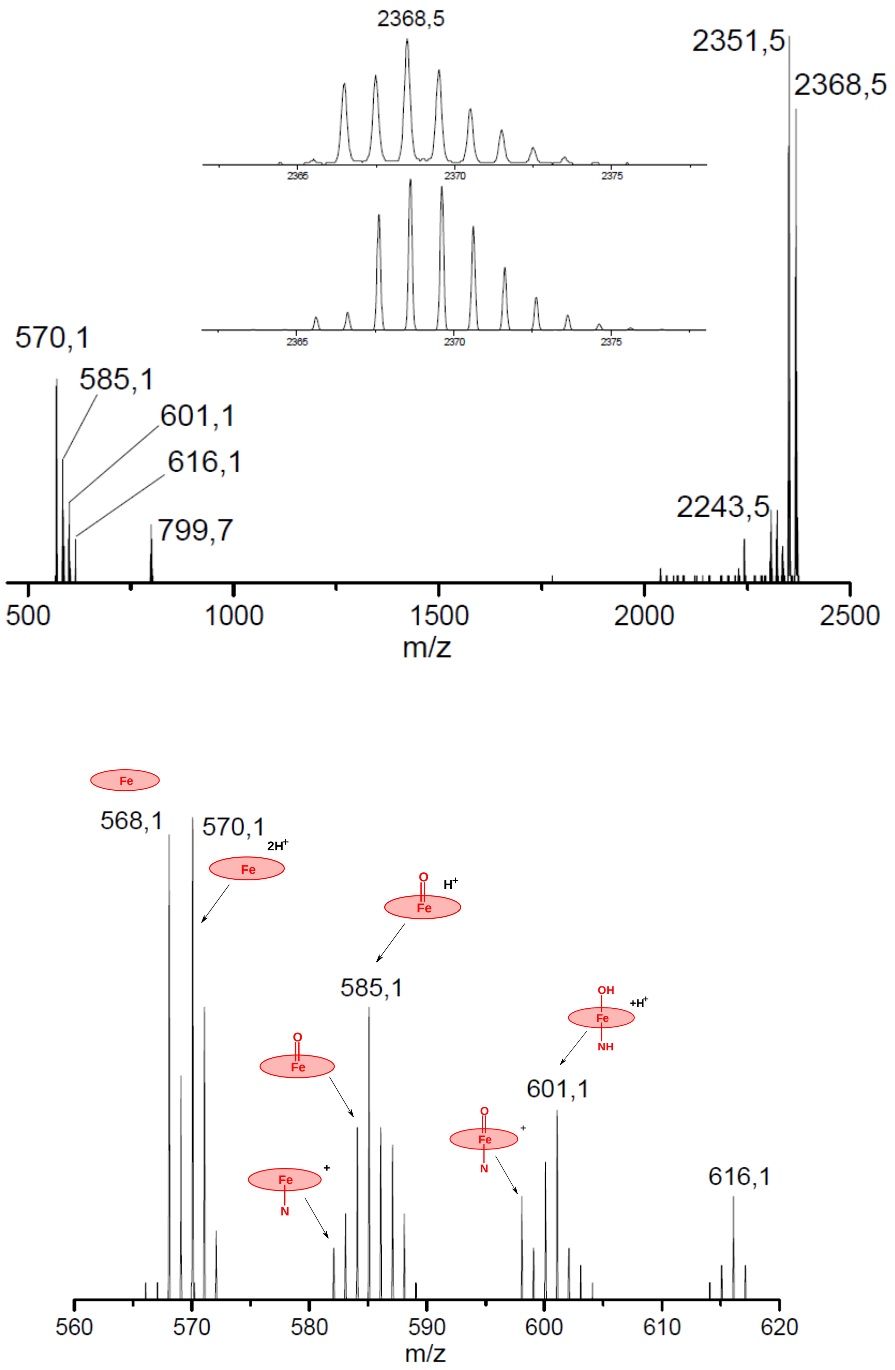

Figure S10. CID MS/MS spectrum of the peroxo complex **7-HOOH** obtained from **7** and H_2O_2. Inset: comparison of experimental (top) and simulated (bottom) isotopic distribution of molecular peak. An enlarged region of spectrum with assignment of fragments is shown below.

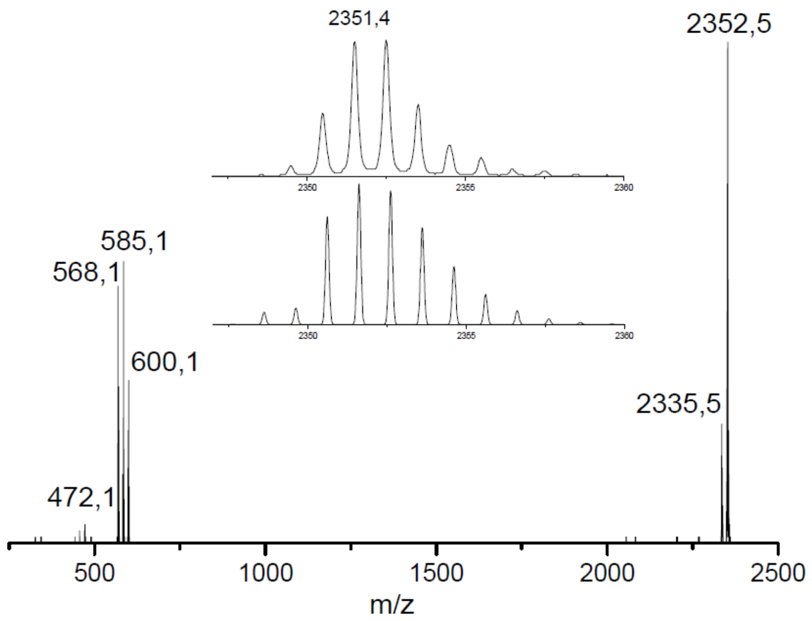

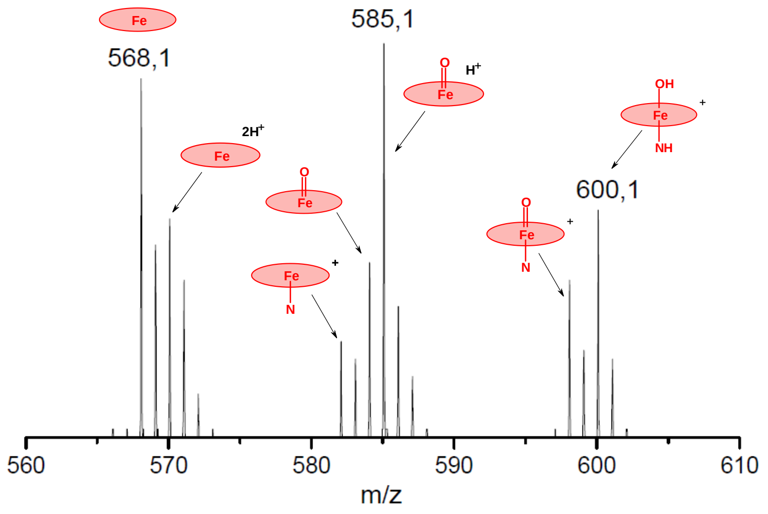

Figure S11. CID MS/MS spectrum of the oxo complex **7=O** obtained from **7** and H_2O_2. Inset: comparison of experimental (top) and simulated (bottom) isotopic distribution of molecular peak. An enlarged region of spectrum with assignment of fragments is shown below.

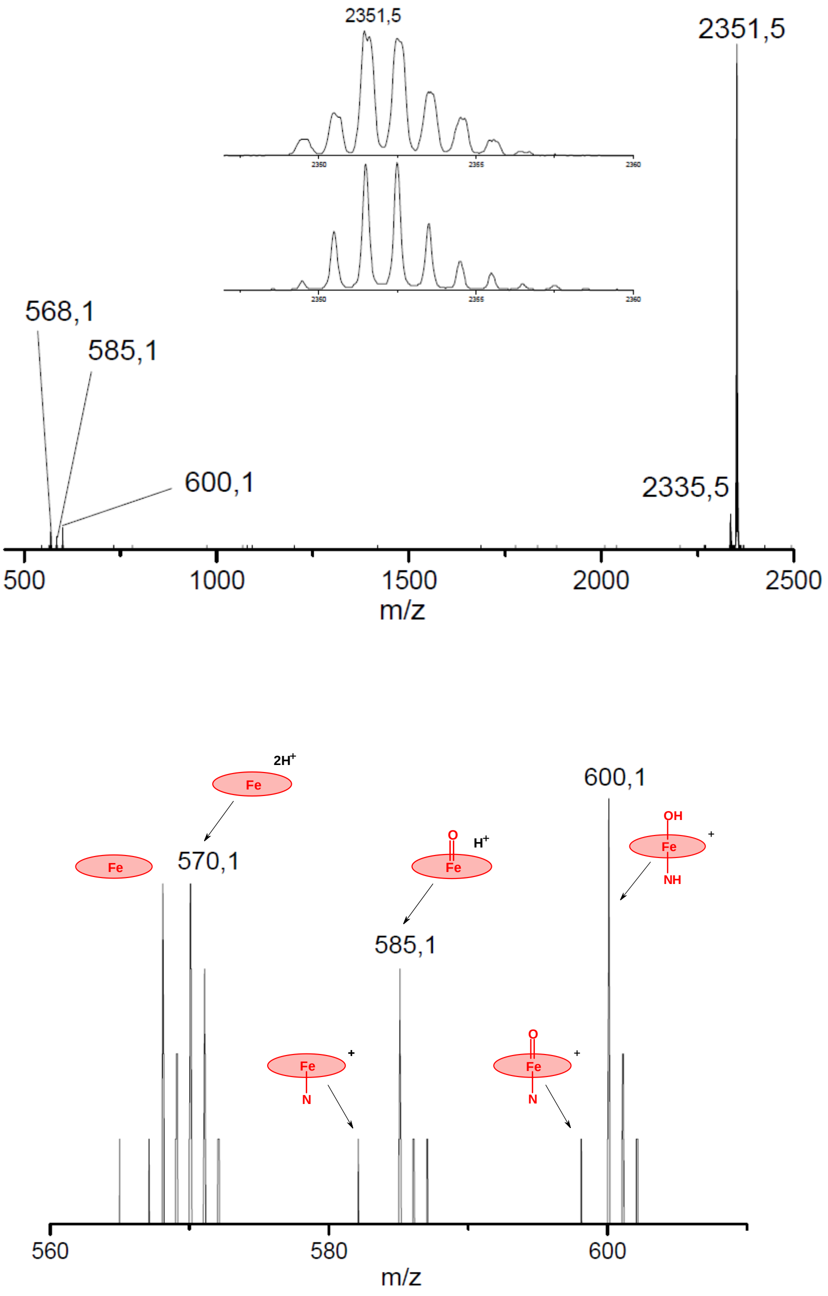

Figure S12. CID MS/MS spectrum of the oxo complex **7=O** obtained from **7** and *m*-CPBA. Inset: comparison of experimental (top) and simulated (bottom) isotopic distribution of molecular peak. An enlarged region of spectrum with assignment of fragments is shown below.

Table S1. Absolute energies (in au) of UB3LYP optimized geometries.

B3LYP	E (au)	ZPE (au)	E+ZPE (au)	G (au)	E_{solv} (au)
^{2}**9**	-5985.81798	0.98363	-5984.83435	-5984.95745	
^{2}[**9**-OOH]$_{B}$	-6136.85161	0.99872	-6135.85289	-6135.97600	-6136.96286
^{4}[**9**-OOH]$_{B}$	-6136.84385	0.99813	-6135.84572	-6135.97267	-6136.95357
^{6}[**9**-OOH]$_{B}$	-6136.83000	0.99752	-6135.83247	-6135.95952	-6136.93869
^{2}[**9**-OOH]$_{A}$	-6136.85665	0.99887	-6135.85778	-6135.98270	-6136.96533
^{4}[**9**-OOH]$_{A}$	-6136.84757	0.99839	-6135.84917	-6135.97249	-6136.95501
^{6}[**9**-OOH]$_{A}$	-6136.79845	0.99415	-6135.80430	-6135.93166	-6136.90784

Table S2. Absolute (in au) and relative (in kcal mol^{-1}) energies of UB3LYP optimized geometries.

	E (au)	ZPE (au)	E+ZPE	G (au)	ΔE+ZPE	ΔG
^{2}[**9**=O]$_{A}$	-6060.94794	0.98506	-6059.96288	-6060.09332	0.00	0.00
^{4}[**9**=O]$_{A}$	-6060.94709	0.98534	-6059.96175	-6060.08635	0.71	4.38
^{6}[**9**=O]$_{A}$	-6060.92568	0.98400	-6059.94168	-6060.07001	13.30	14.63
	E (au)	ZPE (au)	E+ZPE	G (au)		
^{2}[**9**=O]$_{B}$	-6060.94579	0.98534	-6059.96045	-6060.08458	1.52	5.49
^{4}[**9**=O]$_{B}$	-6060.94470	0.98525	-6059.95945	-6060.08402	2.15	5.84
^{6}[**9**=O]$_{B}$	-6060.92860	0.98414	-6059.94447	-6060.07303	11.55	12.73
	E (au)	ZPE (au)	E+ZPE	G (au)		
^{2}**9**	-5985.81798	0.98363	-5984.83435	-5984.95745	0.00	0.00
^{4}**9**	-5985.79786	0.98245	-5984.81541	-5984.93830	11.89	12.01
^{6}**9**	-5985.78993	0.98077	-5984.80916	-5984.93749	15.81	12.53

Table S3. Absolute (in au) and relative (in kcal mol^{-1}) energies of UBP86 optimized geometries.

	E (au)	ZPE (au)	E+ZPE	G (au)	ΔE+ZPE	ΔG
^{2}[**9**=O]$_A$	-6061.58295	0.95566	-6060.62729	-6060.75589	0.00	0.00
^{4}[**9**=O]$_A$	-6061.57498	0.95506	-6060.61992	-6060.75103	4.62	3.05
^{6}[**9**=O]$_A$	-6061.54335	0.95272	-6060.59063	-6060.72172	23.00	21.44
	E (au)	ZPE (au)	E+ZPE	G (au)	ΔE+ZPE	ΔG
^{2}[**9**=O]$_B$	-6061.57913	0.95561	-6060.62352	-6060.74899	2.36	4.33
^{4}[**9**=O]$_B$	-6061.57178	0.95508	-6060.61670	-6060.74522	6.64	6.69
^{6}[**9**=O]$_B$	-6061.54530	0.95287	-6060.59243	-6060.72303	21.87	20.62
	E (au)	ZPE (au)	E+ZPE	G (au)	ΔE+ZPE	ΔG
^{2}**9**	-5986.45635	0.95307	-5985.50328	-5985.63041	0.00	0.00
^{4}**9**	-5986.40680	0.95142	-5985.45538	-5985.58355	30.06	29.40
^{6}**9**	-5986.38221	0.95054	-5985.43167	-5985.56275	44.93	42.46

Table S4. Key bond lengths of UB3LYP optimized geometries with bond lengths in angstroms and angles in degrees.

	^{2}[**9**=O]$_{A}$	^{4}[**9**=O]$_{A}$	^{6}[**9**=O]$_{A}$
Fe-O	1.652	1.648	1.671
N-Fe	2.092	2.201	2.059
Fe-N	1.681	1.679	1.755
Fe-N-Fe	178.8	179.8	172.3
	^{2}[**9**=O]$_{B}$	^{4}[**9**=O]$_{B}$	^{6}[**9**=O]$_{B}$
N-Fe	2.141	2.232	2.049
Fe-N	1.678	1.678	1.749
Fe-O	1.647	1.645	1.670
Fe-N-Fe	179.8	179.8	179.1
	^{2}**9**	^{4}**9**	^{6}**9**
N-Fe	1.668	1.904	1.781
Fe-N	1.653	1.654	1.951
Fe-N-Fe	179.2	170.0	179.0

Table S5. Key bond lengths of UBP86 optimized geometries with bond lengths in angstroms and angles in degrees.

	^{2}[**9**=O]$_{A}$	^{4}[**9**=O]$_{A}$	^{6}[**9**=O]$_{A}$
Fe-O	1.718	1.678	1.687
N-Fe	1.795	2.046	2.075
Fe-N	1.621	1.617	1.681
Fe-N-Fe	179.5	161.9	159.7
	^{2}[**9**=O]$_{B}$	^{4}[**9**=O]$_{B}$	^{6}[**9**=O]$_{B}$
N-Fe	1.627	1.620	1.681
Fe-N	1.788	2.043	2.084
Fe-O	1.714	1.673	1.686
Fe-N-Fe	179.6	163.9	160.5
	^{2}**9**	^{4}**9**	^{6}**9**
N-Fe	1.668	1.904	1.781
Fe-N	1.653	1.654	1.951
Fe-N-Fe	179.2	170.0	179.0

Table S6. Single-point energies (in au) of different fragments calculated at UB3LYP.

Single-point	E (au)
$^{2}[[(PcH)FeOOH]\text{-}N\text{-}(NH_3)_2(NH_2)_2]^{-}$	-2345.40281
$^{2}[(PcH)]\text{-}N\text{-}(NH_3)_2(NH_2)_2$	-2194.32779
$^{2}[[(Pc\mathbf{S})FeOOH]\text{-}N\text{-}(NH_3)_2(NH_2)_2]^{-}$	-4697.10993
$^{2}[(Pc\mathbf{S})]\text{-}N\text{-}(NH_3)_2(NH_2)_2$	-4545.99618
$^{2}[(PcH)FeOOH]$	-1942.03809
$^{2}[(PcS)FeOOH]$	-4293.71064
$^{2}[(PcH)FeOOH]$-(point charge)$]^{-}$	-1942.13658
$^{2}[(PcS)FeOOH]$-(point charge)$]^{-}$	-4293.89695
$^{2}[PcH]^{+}$	-1788.71422
$^{2}[PcS]^{+}$	-4142.41635
$^{2}[PcH]$-(point charge)	-1791.06296
$^{2}[PcS]$-(point charge)	-4142.72038

Table S7. Absolute energies of UBP86 optimized geometries.

BP86	E (au)	ZPE (au)	E+ZPE (au)	G (au)
$^{2}[\mathbf{9}\text{-OOH}]_A$	-6137.48876	0.96675	-6136.52201	-6136.64950
$^{2}[\mathbf{9}\text{-OOH}]_B$	-6137.49056	0.96726	-6136.52331	-6136.65230

Cartesian coordinates

Structure ^{2}[**9**=O]$_{A}$

26	-1.780091000	-0.974714000	2.079407000
7	-2.882806000	0.064395000	0.831610000
7	-0.739898000	0.646660000	2.451888000
7	-3.129088000	-2.399196000	2.046091000
7	-0.981408000	-1.820005000	3.661120000
6	0.314937000	0.749727000	3.356928000
6	-2.628836000	1.359450000	0.383159000
6	-0.810814000	1.852980000	1.757003000
6	-4.187220000	-2.500247000	1.144821000
6	0.107946000	-1.336402000	4.382266000
6	-3.119603000	-3.566102000	2.808414000
6	-1.297303000	-3.075556000	4.177248000
6	-4.442141000	0.605239000	-0.807812000
6	0.932049000	2.060304000	3.238793000
6	-3.593742000	1.719395000	-0.641116000
6	0.222495000	2.753589000	2.236420000
6	-4.876160000	-3.764765000	1.341682000
6	0.495859000	-2.308575000	5.390528000
6	-4.207581000	-4.431736000	2.389159000
6	-0.386975000	-3.400794000	5.261329000
26	0.552798000	-2.415344000	-0.511986000
7	0.494750000	-0.646846000	-1.388940000
7	-1.063449000	-2.953956000	-1.506957000
7	2.043001000	-1.800036000	0.625177000
7	0.498827000	-4.115136000	0.490925000
6	1.393263000	0.382937000	-1.180158000
6	-1.685509000	-4.187238000	-1.423546000
6	-0.373820000	-0.276269000	-2.399356000
6	-1.687201000	-2.210963000	-2.493505000
6	2.693101000	-0.584083000	0.509405000
6	-0.376811000	-5.159086000	0.256266000
6	2.697040000	-2.561018000	1.577848000
6	1.399890000	-4.505574000	1.464755000
6	1.084689000	1.476124000	-2.094193000
6	-2.771189000	-4.242096000	-2.395716000
6	-0.023604000	1.060844000	-2.866065000
6	-2.775144000	-2.996422000	-3.062721000
6	3.816050000	-0.551474000	1.439075000
6	-0.031046000	-6.275000000	1.128531000
6	3.814946000	-1.793477000	2.113001000
6	1.090737000	-5.867971000	1.885240000
7	-0.738318000	-1.633018000	0.936152000
7	0.710236000	-0.164874000	4.231423000
7	-4.567113000	-1.595592000	0.253241000
7	2.392700000	0.419226000	-0.306760000

7	-1.380158000	-5.193422000	-0.612717000
6	-3.980184000	-0.414211000	0.119204000
8	1.569730000	-3.032359000	-1.657968000
7	-2.274721000	-3.878741000	3.780927000
7	-1.379283000	-0.985086000	-2.899756000
7	-1.673513000	2.176691000	0.803869000
7	2.401372000	-3.794460000	1.970005000
6	-0.588796000	-7.545084000	1.283882000
6	1.702481000	-6.721530000	2.807072000
6	1.156447000	-7.998665000	2.974716000
6	0.025726000	-8.358294000	2.228567000
6	-3.701005000	-2.715776000	-4.069311000
6	-3.677545000	-5.253653000	-2.724614000
6	-4.611664000	-4.989135000	-3.731606000
6	-4.605808000	-3.731779000	-4.351749000
6	1.679756000	2.723222000	-2.291582000
6	1.102868000	3.513094000	-3.278850000
6	-0.562545000	1.869271000	-3.870323000
6	0.022475000	3.122362000	-4.081867000
6	4.780069000	-2.096521000	3.075390000
6	5.714452000	-1.098098000	3.322315000
6	4.787447000	0.420662000	1.691731000
6	5.761274000	0.133262000	2.653798000
6	0.560275000	4.062858000	1.883364000
6	2.000926000	2.656894000	3.912649000
6	2.343546000	3.964057000	3.555669000
6	1.631422000	4.659318000	2.553734000
6	-3.776596000	2.886940000	-1.387241000
6	-4.827296000	2.909259000	-2.308362000
6	-5.677266000	1.793933000	-2.474757000
6	-5.496655000	0.629041000	-1.723826000
6	-5.987698000	-4.333787000	0.714790000
6	-6.411462000	-5.588738000	1.160901000
6	-5.743838000	-6.255356000	2.211704000
6	-4.634581000	-5.683612000	2.840392000
6	-0.288864000	-4.514893000	6.099441000
6	0.718164000	-4.509869000	7.068343000
6	1.600859000	-3.415399000	7.199069000
6	1.498562000	-2.299723000	6.363427000
16	-0.690554000	-10.073840000	2.489155000
16	-5.882889000	-3.405417000	-5.688196000
16	1.854125000	5.206634000	-3.580874000
8	-5.332117000	-2.150059000	-6.596978000
8	-6.294544000	-4.850984000	-6.354882000
8	-1.600378000	-10.432094000	1.166740000
8	0.525329000	-11.054197000	3.003218000
8	8.357629000	-0.547771000	4.226208000
8	7.112936000	-3.075911000	4.808717000
8	3.368514000	5.194506000	-2.939478000

8	1.574373000	5.608585000	-5.150596000
1	-5.314962000	-5.745462000	-4.062544000
1	-3.644828000	-6.214350000	-2.225093000
1	-3.708926000	-1.780437000	-4.616761000
1	-1.429572000	-7.894290000	0.695784000
1	1.608671000	-8.723213000	3.643042000
1	2.579888000	-6.407700000	3.359550000
1	4.824065000	-3.053159000	3.582931000
1	4.792825000	1.356920000	1.146844000
1	6.567968000	0.826065000	2.866838000
1	2.549222000	3.054496000	-1.735777000
1	-0.320715000	3.779953000	-4.873062000
1	-1.394912000	1.527378000	-4.473421000
1	-6.148443000	-0.229274000	-1.838639000
1	-6.483507000	1.847641000	-3.198647000
1	-4.997784000	3.797583000	-2.907313000
1	-3.123073000	3.740275000	-1.250145000
1	-6.497946000	-3.814242000	-0.087570000
1	-7.269743000	-6.062105000	0.695845000
1	-6.102740000	-7.227333000	2.533201000
1	-4.119743000	-6.183813000	3.652473000
1	-0.973933000	-5.348056000	5.996013000
1	0.826086000	-5.358624000	7.735344000
1	2.369354000	-3.445184000	7.964275000
1	2.166560000	-1.451920000	6.461746000
1	2.540206000	2.118755000	4.683155000
1	3.169749000	4.458095000	4.055978000
1	1.924744000	5.673562000	2.304400000
1	0.002835000	4.589950000	1.117728000
16	7.034539000	-1.445123000	4.611181000
6	-7.403331000	-2.780057000	-4.741369000
1	-8.157364000	-2.583369000	-5.502252000
1	-7.709558000	-3.575131000	-4.063592000
1	-7.103357000	-1.871740000	-4.221412000
6	-1.886897000	-9.866313000	3.946807000
1	-2.317695000	-10.853212000	4.110069000
1	-1.297977000	-9.535117000	4.800517000
1	-2.638260000	-9.139663000	3.642142000
6	0.811063000	6.379250000	-2.515151000
1	1.234023000	7.369861000	-2.676407000
1	-0.214718000	6.313471000	-2.874011000
1	0.920992000	6.053290000	-1.482181000
6	6.326460000	-0.725798000	6.217566000
1	7.082479000	-0.917787000	6.977705000
1	6.177621000	0.340366000	6.054727000
1	5.398890000	-1.257458000	6.423361000

Structure $^{2}[\mathbf{9}{=}O]_{B}$

26	-1.969382000	-0.795423000	2.224677000

7	-3.099538000	-0.184697000	0.725575000
7	-0.911035000	0.863380000	2.095362000
7	-2.881342000	-2.544067000	2.190437000
7	-0.707882000	-1.486327000	3.577303000
6	0.151013000	1.209502000	2.915658000
6	-3.045679000	1.064523000	0.130919000
6	-1.210920000	1.949244000	1.288148000
6	-3.995259000	-2.858154000	1.428367000
6	0.318882000	-0.765582000	4.164225000
6	-2.630426000	-3.600132000	3.052327000
6	-0.795627000	-2.715045000	4.209346000
6	-4.850878000	-0.030467000	-0.784387000
6	0.563005000	2.575070000	2.615098000
6	-4.134851000	1.185460000	-0.830950000
6	-0.296329000	3.041355000	1.596519000
6	-4.477264000	-4.180582000	1.807008000
6	0.921853000	-1.566648000	5.222856000
6	-3.623196000	-4.644154000	2.831224000
6	0.226043000	-2.794938000	5.246635000
26	0.392537000	-2.239487000	-0.405788000
7	0.722495000	-0.580841000	-1.397354000
7	-0.986093000	-2.710834000	-1.726886000
7	2.063039000	-1.947056000	0.590492000
7	0.380416000	-4.093390000	0.232444000
6	1.686514000	0.374977000	-1.087764000
6	-1.713465000	-3.895674000	-1.758748000
6	-0.046836000	-0.077154000	-2.443170000
6	-1.496594000	-1.878676000	-2.717180000
6	2.819088000	-0.779737000	0.591043000
6	-0.559378000	-5.064043000	-0.104325000
6	2.636012000	-2.818206000	1.510436000
6	1.213868000	-4.636861000	1.206664000
6	1.531575000	1.520533000	-1.967984000
6	-2.725130000	-3.820917000	-2.801725000
6	0.446224000	1.235881000	-2.823642000
6	-2.589331000	-2.549944000	-3.399977000
6	3.912259000	-0.905247000	1.543083000
6	-0.312812000	-6.266192000	0.673602000
6	3.792414000	-2.186651000	2.122226000
6	0.803512000	-6.000880000	1.494952000
7	-0.646465000	-1.602761000	0.748033000
7	0.720829000	0.462845000	3.855683000
7	-4.583678000	-2.100089000	0.509108000
7	2.636573000	0.289956000	-0.168925000
7	-1.525160000	-4.969015000	-1.005392000
6	-4.178535000	-0.873630000	0.197063000
7	-1.668785000	-3.686793000	3.964909000
7	-1.070254000	-0.667435000	-3.041856000
7	-2.176261000	2.038509000	0.379625000

7	2.242047000	-4.049298000	1.799651000
6	-0.950612000	-7.508728000	0.692752000
6	1.331742000	-6.969625000	2.354027000
6	0.703130000	-8.217774000	2.387417000
6	-0.419435000	-8.438843000	1.576064000
6	-3.406474000	-2.154362000	-4.461143000
6	-3.670815000	-4.747256000	-3.250618000
6	-4.499963000	-4.365411000	-4.310097000
6	-4.354451000	-3.085549000	-4.864062000
6	2.248553000	2.714453000	-2.075746000
6	1.809821000	3.587156000	-3.062342000
6	0.047072000	2.124715000	-3.826705000
6	0.752681000	3.325478000	-3.946309000
6	4.705307000	-2.642986000	3.075340000
6	5.711990000	-1.748607000	3.414413000
6	4.955308000	-0.039512000	1.884913000
6	5.875480000	-0.479135000	2.841948000
6	-0.190280000	4.343234000	1.101268000
6	1.556874000	3.395250000	3.154393000
6	1.667777000	4.696957000	2.656165000
6	0.801014000	5.166875000	1.645660000
6	-4.533462000	2.228003000	-1.670401000
6	-5.671538000	2.030159000	-2.458530000
6	-6.394013000	0.818756000	-2.403705000
6	-5.990024000	-0.228362000	-1.567900000
6	-5.560463000	-4.945122000	1.367216000
6	-5.775145000	-6.183013000	1.980694000
6	-4.927806000	-6.641669000	3.012603000
6	-3.838973000	-5.878696000	3.448335000
6	0.550370000	-3.792461000	6.168668000
6	1.583258000	-3.530914000	7.074337000
6	2.271112000	-2.298421000	7.058430000
6	1.951076000	-1.302129000	6.129307000
16	-1.234314000	-10.130035000	1.645646000
16	-5.484198000	-2.608308000	-6.286847000
16	2.737093000	5.209280000	-3.257905000
16	6.969660000	-2.306221000	4.693162000
8	-4.728595000	-1.407911000	-7.119075000
8	-5.971019000	-4.001406000	-7.012272000
8	-2.049591000	-10.330476000	0.231637000
8	-0.094721000	-11.200036000	2.155709000
8	8.350202000	-1.454602000	4.423400000
8	6.946016000	-3.950531000	4.706739000
8	4.225778000	4.996156000	-2.592522000
8	2.538079000	5.700175000	-4.814600000
1	-5.229886000	-5.047537000	-4.732134000
1	-3.747796000	-5.729319000	-2.800148000
1	-3.302062000	-1.197315000	-4.958768000
1	-1.789125000	-7.748585000	0.049513000

1	1.082843000	-9.026540000	3.002117000
1	2.204621000	-6.761348000	2.960652000
1	4.654386000	-3.634532000	3.509773000
1	5.052215000	0.931365000	1.414461000
1	6.728993000	0.126331000	3.126831000
1	3.104551000	2.943894000	-1.452040000
1	0.521149000	4.042951000	-4.725885000
1	-0.771294000	1.882350000	-4.493677000
1	-6.545676000	-1.157403000	-1.509068000
1	-7.289743000	0.710396000	-3.007758000
1	-6.013636000	2.822364000	-3.116269000
1	-3.980401000	3.159668000	-1.696996000
1	-6.214705000	-4.579252000	0.584445000
1	-6.612008000	-6.799079000	1.668666000
1	-5.141575000	-7.594866000	3.486580000
1	-3.191268000	-6.215674000	4.249751000
1	0.007904000	-4.730635000	6.182927000
1	1.855655000	-4.280908000	7.809551000
1	3.047765000	-2.117545000	7.795338000
1	2.465139000	-0.347661000	6.117957000
1	2.209175000	3.031075000	3.939559000
1	2.424274000	5.363032000	3.057778000
1	0.897014000	6.192651000	1.302878000
1	-0.869649000	4.699090000	0.334985000
8	-2.987412000	-0.175450000	3.361413000
6	1.793500000	6.426526000	-2.151693000
1	2.354927000	7.358451000	-2.203138000
1	0.795151000	6.530518000	-2.573692000
1	1.787909000	6.006137000	-1.147074000
6	-7.001805000	-1.843816000	-5.445317000
1	-7.641733000	-1.502528000	-6.257921000
1	-7.472758000	-2.633706000	-4.862310000
1	-6.644320000	-1.019000000	-4.830548000
6	-2.526284000	-9.974054000	3.024876000
1	-3.047891000	-10.930068000	3.041040000
1	-1.982448000	-9.793073000	3.950674000
1	-3.186147000	-9.151467000	2.752804000
6	6.250363000	-1.719429000	6.346702000
1	6.930211000	-2.098868000	7.108352000
1	6.238345000	-0.631013000	6.319283000
1	5.254790000	-2.152380000	6.433153000

Structure ^{2}**9**

26	-1.642312000	-1.068447000	2.077502000
7	-2.891625000	-0.367210000	0.734053000
7	-0.883791000	0.742082000	2.264561000
7	-2.779407000	-2.659289000	2.258199000
7	-0.761125000	-1.558870000	3.795382000
6	0.102944000	1.128376000	3.167628000

6	-2.846278000	0.894337000	0.147103000
6	-1.128887000	1.823564000	1.425641000
6	-3.843668000	-3.008890000	1.432636000
6	0.215455000	-0.821423000	4.449712000
6	-2.591351000	-3.702267000	3.162809000
6	-0.866988000	-2.781614000	4.442686000
6	-4.624386000	-0.200499000	-0.810277000
6	0.498826000	2.503012000	2.897667000
6	-3.915609000	1.018014000	-0.830991000
6	-0.278781000	2.941117000	1.806170000
6	-4.348512000	-4.318938000	1.813566000
6	0.753190000	-1.595847000	5.559842000
6	-3.565274000	-4.751716000	2.903166000
6	0.073830000	-2.832384000	5.553479000
26	0.386952000	-2.323558000	-0.219984000
7	0.637645000	-0.651094000	-1.218518000
7	-1.079085000	-2.800387000	-1.439382000
7	2.173621000	-2.044810000	0.613315000
7	0.444231000	-4.199551000	0.389548000
6	1.631391000	0.295334000	-0.989294000
6	-1.758435000	-4.012960000	-1.472354000
6	-0.201238000	-0.151967000	-2.206780000
6	-1.630831000	-1.983426000	-2.418721000
6	2.910928000	-0.871807000	0.577229000
6	-0.497593000	-5.178471000	0.106473000
6	2.793833000	-2.909044000	1.500253000
6	1.352540000	-4.735319000	1.295180000
6	1.409213000	1.446129000	-1.852195000
6	-2.784638000	-3.972045000	-2.504167000
6	0.260287000	1.164869000	-2.621966000
6	-2.702872000	-2.695110000	-3.099073000
6	4.052507000	-0.982033000	1.476931000
6	-0.188253000	-6.383891000	0.860617000
6	3.972696000	-2.265121000	2.061959000
6	0.978967000	-6.109060000	1.605113000
7	-0.452893000	-1.709822000	1.110726000
7	0.619484000	0.407678000	4.154095000
7	-4.382527000	-2.273203000	0.469265000
7	2.656161000	0.206501000	-0.152992000
7	-1.512742000	-5.091861000	-0.741773000
6	-3.962935000	-1.051267000	0.167273000
7	-1.701664000	-3.769456000	4.144506000
7	-1.245438000	-0.759870000	-2.753196000
7	-2.019638000	1.888327000	0.445116000
7	2.414549000	-4.139699000	1.819214000
6	-0.810797000	-7.632156000	0.919344000
6	1.573632000	-7.075574000	2.421186000
6	0.959604000	-8.329948000	2.496758000
6	-0.213496000	-8.559291000	1.764044000

6	-3.527580000	-2.338096000	-4.168106000
6	-3.684765000	-4.939497000	-2.960127000
6	-4.521055000	-4.597343000	-4.027175000
6	-4.426845000	-3.312911000	-4.581716000
6	2.118501000	2.637847000	-2.014775000
6	1.604095000	3.516115000	-2.960064000
6	-0.212735000	2.056610000	-3.588830000
6	0.480916000	3.258937000	-3.758975000
6	4.916441000	-2.699075000	2.993912000
6	5.911351000	-1.782100000	3.309913000
6	5.089453000	-0.097977000	1.788193000
6	6.039569000	-0.513839000	2.727338000
6	-0.166272000	4.243908000	1.313551000
6	1.417371000	3.352562000	3.519777000
6	1.538121000	4.653496000	3.022816000
6	0.752557000	5.095104000	1.935614000
6	-4.311564000	2.073719000	-1.656308000
6	-5.439122000	1.881805000	-2.459232000
6	-6.157755000	0.666551000	-2.427983000
6	-5.758628000	-0.390606000	-1.604270000
6	-5.396893000	-5.099736000	1.319576000
6	-5.643811000	-6.325230000	1.944588000
6	-4.865885000	-6.754076000	3.042609000
6	-3.816497000	-5.972278000	3.535712000
6	0.349894000	-3.814767000	6.507093000
6	1.326164000	-3.531443000	7.467564000
6	2.002993000	-2.292736000	7.476656000
6	1.723448000	-1.308271000	6.522847000
16	-1.017487000	-10.249769000	1.901239000
16	-5.549180000	-2.901182000	-6.029613000
16	2.507364000	5.141851000	-3.215604000
16	7.179546000	-2.292779000	4.595413000
8	-4.847327000	-1.664794000	-6.856172000
8	-5.953427000	-4.318679000	-6.758905000
8	-1.921619000	-10.475767000	0.546197000
8	0.147099000	-11.316672000	2.359119000
8	8.535467000	-1.398585000	4.337891000
8	7.212502000	-3.936735000	4.627847000
8	4.057191000	4.928476000	-2.708934000
8	2.150706000	5.670975000	-4.731306000
1	-5.211490000	-5.314119000	-4.458057000
1	-3.716180000	-5.925753000	-2.513111000
1	-3.458522000	-1.378762000	-4.667298000
1	-1.688884000	-7.877644000	0.333542000
1	1.386609000	-9.135375000	3.084188000
1	2.485281000	-6.861253000	2.965714000
1	4.892416000	-3.688702000	3.435282000
1	5.156508000	0.873189000	1.312796000
1	6.884686000	0.111097000	2.994517000

1	3.023717000	2.860698000	-1.462132000
1	0.189716000	3.980361000	-4.514530000
1	-1.079241000	1.815613000	-4.192652000
1	-6.310131000	-1.322940000	-1.564657000
1	-7.044798000	0.563858000	-3.045395000
1	-5.776575000	2.680026000	-3.111844000
1	-3.762244000	3.007825000	-1.662696000
1	-5.995117000	-4.758484000	0.482888000
1	-6.449700000	-6.957629000	1.587390000
1	-5.100563000	-7.701896000	3.517033000
1	-3.221032000	-6.286958000	4.385132000
1	-0.179222000	-4.760566000	6.498323000
1	1.566453000	-4.272200000	8.223017000
1	2.745097000	-2.101075000	8.245217000
1	2.230686000	-0.350229000	6.528742000
1	2.008198000	3.008983000	4.360798000
1	2.240871000	5.340607000	3.482274000
1	0.857776000	6.118753000	1.589881000
1	-0.782783000	4.577258000	0.486572000
6	-2.223719000	-10.072662000	3.354020000
1	-2.733206000	-11.032535000	3.426632000
1	-1.626552000	-9.862277000	4.239829000
1	-2.908061000	-9.263799000	3.101928000
6	-7.124375000	-2.213771000	-5.229433000
1	-7.776787000	-1.944832000	-6.059022000
1	-7.549177000	-3.011187000	-4.621769000
1	-6.827696000	-1.345088000	-4.644140000
6	1.680727000	6.333315000	-1.993074000
1	0.641503000	6.434972000	-2.301697000
1	1.788044000	5.897792000	-1.000616000
1	2.226172000	7.271116000	-2.089474000
6	6.424962000	-1.717300000	6.238827000
1	6.367245000	-0.630784000	6.198437000
1	5.447637000	-2.190092000	6.326265000
1	7.114630000	-2.059138000	7.009342000

^{2}[(Pc**S**)FeOOH]-N-$(NH_3)_2(NH_2)_2$

26	-1.778414000	-0.982293000	2.069019000
7	-3.105838000	-0.186639000	0.840327000
7	-0.899348000	0.772705000	2.212246000
7	-2.920931000	-2.574795000	2.229266000
7	-0.714281000	-1.616104000	3.594463000
1	-0.106394000	1.000523000	2.812015000
1	-3.297260000	0.740920000	1.161249000
1	-1.110075000	1.597516000	1.650391000
1	-3.753983000	-2.775933000	1.676123000
1	-0.468354000	-0.938621000	4.287670000
1	-2.755788000	-3.373446000	2.841870000
1	-1.240784000	-2.437048000	3.815475000

26	0.455952000	-2.358790000	-0.430282000
7	0.586852000	-0.619482000	-1.343958000
7	-1.062130000	-2.836144000	-1.589093000
7	2.012263000	-1.906374000	0.703663000
7	0.365389000	-4.122851000	0.456402000
6	1.537786000	0.341889000	-1.082899000
6	-1.727993000	-4.042894000	-1.567894000
6	-0.214989000	-0.182006000	-2.375509000
6	-1.604098000	-2.046799000	-2.578539000
6	2.732434000	-0.732538000	0.622994000
6	-0.531520000	-5.120288000	0.135218000
6	2.598160000	-2.720309000	1.649969000
6	1.206590000	-4.586676000	1.446430000
6	1.329898000	1.474770000	-1.981815000
6	-2.774377000	-4.032142000	-2.588089000
6	0.225725000	1.144466000	-2.799578000
6	-2.696444000	-2.771126000	-3.221837000
6	3.840167000	-0.780852000	1.573902000
6	-0.264690000	-6.288409000	0.970311000
6	3.753870000	-2.034668000	2.220970000
6	0.830986000	-5.952894000	1.798086000
7	-0.740902000	-1.605783000	0.809346000
7	2.515122000	0.301596000	-0.183371000
7	-1.494025000	-5.088259000	-0.780640000
1	-3.850751000	-0.819007000	0.627685000
8	1.656132000	-3.130891000	-1.609960000
7	-1.232603000	-0.823561000	-2.938167000
7	2.228328000	-3.945832000	2.006256000
6	-0.868920000	-7.543418000	1.041728000
6	1.361556000	-6.863807000	2.714389000
6	0.763332000	-8.125194000	2.806514000
6	-0.332598000	-8.416272000	1.982938000
6	-3.566255000	-2.423796000	-4.254726000
6	-3.714673000	-4.988690000	-2.976539000
6	-4.602722000	-4.655298000	-4.005503000
6	-4.509574000	-3.387975000	-4.595800000
6	1.997965000	2.690499000	-2.122254000
6	1.493468000	3.543689000	-3.098699000
6	-0.236206000	2.012669000	-3.790794000
6	0.415732000	3.241743000	-3.941613000
6	4.682047000	-2.414555000	3.190416000
6	5.668238000	-1.476418000	3.478635000
6	4.859300000	0.127054000	1.869630000
6	5.794583000	-0.232342000	2.846243000
16	-1.132273000	-10.098925000	2.169182000
16	-5.754552000	-2.958837000	-5.926642000
16	2.301328000	5.221400000	-3.293957000
8	-5.067963000	-1.897053000	-6.979408000
8	-6.437181000	-4.359510000	-6.460497000

8	-1.793603000	-10.537486000	0.727746000
8	-0.071643000	-11.087100000	2.951046000
8	8.209197000	-0.892056000	4.633691000
8	7.119633000	-3.533132000	4.849907000
8	3.881270000	5.108981000	-2.848511000
8	1.876527000	5.827075000	-4.765497000
1	-5.344520000	-5.357259000	-4.369692000
1	-3.749792000	-5.959156000	-2.495956000
1	-3.501402000	-1.471876000	-4.768552000
1	-1.689705000	-7.827427000	0.393681000
1	1.139951000	-8.887890000	3.478872000
1	2.215165000	-6.600034000	3.327384000
1	4.649532000	-3.384343000	3.672862000
1	4.924565000	1.075598000	1.349998000
1	6.625211000	0.413791000	3.107306000
1	2.862775000	2.948091000	-1.522236000
1	0.123142000	3.953685000	-4.705266000
1	-1.070255000	1.736694000	-4.424955000
16	6.918548000	-1.900708000	4.806330000
6	-7.100223000	-2.006086000	-4.991745000
1	-7.809212000	-1.679536000	-5.751171000
1	-7.549548000	-2.704783000	-4.287776000
1	-6.614266000	-1.166976000	-4.495622000
6	-2.575023000	-9.786094000	3.357432000
1	-3.098298000	-10.737651000	3.439237000
1	-2.140267000	-9.476923000	4.306665000
1	-3.196875000	-9.012195000	2.909682000
6	1.440534000	6.289136000	-1.985879000
1	1.926498000	7.262433000	-2.036104000
1	0.389399000	6.343222000	-2.264856000
1	1.593592000	5.802618000	-1.023502000
6	6.049230000	-1.417296000	6.419125000
1	6.730515000	-1.707866000	7.217505000
1	5.896426000	-0.339734000	6.385497000
1	5.112941000	-1.972328000	6.455848000
8	1.417216000	-2.725924000	-3.055502000
1	2.068834000	-3.322612000	-3.485586000
1	-2.633395000	-0.138235000	-0.039704000
1	0.114623000	-2.062331000	3.257120000

^{2}[(Pc**S**)FeOOH]

26	0.455952000	-2.358790000	-0.430282000
7	0.586852000	-0.619482000	-1.343958000
7	-1.062130000	-2.836144000	-1.589093000
7	2.012263000	-1.906374000	0.703663000
7	0.365389000	-4.122851000	0.456402000
6	1.537786000	0.341889000	-1.082899000
6	-1.727993000	-4.042894000	-1.567894000
6	-0.214989000	-0.182006000	-2.375509000

6	-1.604098000	-2.046799000	-2.578539000
6	2.732434000	-0.732538000	0.622994000
6	-0.531520000	-5.120288000	0.135218000
6	2.598160000	-2.720309000	1.649969000
6	1.206590000	-4.586676000	1.446430000
6	1.329898000	1.474770000	-1.981815000
6	-2.774377000	-4.032142000	-2.588089000
6	0.225725000	1.144466000	-2.799578000
6	-2.696444000	-2.771126000	-3.221837000
6	3.840167000	-0.780852000	1.573902000
6	-0.264690000	-6.288409000	0.970311000
6	3.753870000	-2.034668000	2.220970000
6	0.830986000	-5.952894000	1.798086000
7	2.515122000	0.301596000	-0.183371000
7	-1.494025000	-5.088259000	-0.780640000
8	1.656132000	-3.130891000	-1.609960000
7	-1.232603000	-0.823561000	-2.938167000
7	2.228328000	-3.945832000	2.006256000
6	-0.868920000	-7.543418000	1.041728000
6	1.361556000	-6.863807000	2.714389000
6	0.763332000	-8.125194000	2.806514000
6	-0.332598000	-8.416272000	1.982938000
6	-3.566255000	-2.423796000	-4.254726000
6	-3.714673000	-4.988690000	-2.976539000
6	-4.602722000	-4.655298000	-4.005503000
6	-4.509574000	-3.387975000	-4.595800000
6	1.997965000	2.690499000	-2.122254000
6	1.493468000	3.543689000	-3.098699000
6	-0.236206000	2.012669000	-3.790794000
6	0.415732000	3.241743000	-3.941613000
6	4.682047000	-2.414555000	3.190416000
6	5.668238000	-1.476418000	3.478635000
6	4.859300000	0.127054000	1.869630000
6	5.794583000	-0.232342000	2.846243000
16	-1.132273000	-10.098925000	2.169182000
16	-5.754552000	-2.958837000	-5.926642000
16	2.301328000	5.221400000	-3.293957000
8	-5.067963000	-1.897053000	-6.979408000
8	-6.437181000	-4.359510000	-6.460497000
8	-1.793603000	-10.537486000	0.727746000
8	-0.071643000	-11.087100000	2.951046000
8	8.209197000	-0.892056000	4.633691000
8	7.119633000	-3.533132000	4.849907000
8	3.881270000	5.108981000	-2.848511000
8	1.876527000	5.827075000	-4.765497000
1	-5.344520000	-5.357259000	-4.369692000
1	-3.749792000	-5.959156000	-2.495956000
1	-3.501402000	-1.471876000	-4.768552000
1	-1.689705000	-7.827427000	0.393681000

1	1.139951000	-8.887890000	3.478872000
1	2.215165000	-6.600034000	3.327384000
1	4.649532000	-3.384343000	3.672862000
1	4.924565000	1.075598000	1.349998000
1	6.625211000	0.413791000	3.107306000
1	2.862775000	2.948091000	-1.522236000
1	0.123142000	3.953685000	-4.705266000
1	-1.070255000	1.736694000	-4.424955000
16	6.918548000	-1.900708000	4.806330000
6	-7.100223000	-2.006086000	-4.991745000
1	-7.809212000	-1.679536000	-5.751171000
1	-7.549548000	-2.704783000	-4.287776000
1	-6.614266000	-1.166976000	-4.495622000
6	-2.575023000	-9.786094000	3.357432000
1	-3.098298000	-10.737651000	3.439237000
1	-2.140267000	-9.476923000	4.306665000
1	-3.196875000	-9.012195000	2.909682000
6	1.440534000	6.289136000	-1.985879000
1	1.926498000	7.262433000	-2.036104000
1	0.389399000	6.343222000	-2.264856000
1	1.593592000	5.802618000	-1.023502000
6	6.049230000	-1.417296000	6.419125000
1	6.730515000	-1.707866000	7.217505000
1	5.896426000	-0.339734000	6.385497000
1	5.112941000	-1.972328000	6.455848000
8	1.417216000	-2.725924000	-3.055502000
1	2.068834000	-3.322612000	-3.485586000

^{2}[(Pc**S**)]-N-$(NH_3)_2(NH_2)_2$

26	-1.778414000	-0.982293000	2.069019000
7	-3.105838000	-0.186639000	0.840327000
7	-0.899348000	0.772705000	2.212246000
7	-2.920931000	-2.574795000	2.229266000
7	-0.714281000	-1.616104000	3.594463000
1	-0.106394000	1.000523000	2.812015000
1	-3.297260000	0.740920000	1.161249000
1	-1.110075000	1.597516000	1.650391000
1	-3.753983000	-2.775933000	1.676123000
1	-0.468354000	-0.938621000	4.287670000
1	-2.755788000	-3.373446000	2.841870000
1	-1.240784000	-2.437048000	3.815475000
26	0.455952000	-2.358790000	-0.430282000
7	0.586852000	-0.619482000	-1.343958000
7	-1.062130000	-2.836144000	-1.589093000
7	2.012263000	-1.906374000	0.703663000
7	0.365389000	-4.122851000	0.456402000
6	1.537786000	0.341889000	-1.082899000
6	-1.727993000	-4.042894000	-1.567894000
6	-0.214989000	-0.182006000	-2.375509000

6	-1.604098000	-2.046799000	-2.578539000
6	2.732434000	-0.732538000	0.622994000
6	-0.531520000	-5.120288000	0.135218000
6	2.598160000	-2.720309000	1.649969000
6	1.206590000	-4.586676000	1.446430000
6	1.329898000	1.474770000	-1.981815000
6	-2.774377000	-4.032142000	-2.588089000
6	0.225725000	1.144466000	-2.799578000
6	-2.696444000	-2.771126000	-3.221837000
6	3.840167000	-0.780852000	1.573902000
6	-0.264690000	-6.288409000	0.970311000
6	3.753870000	-2.034668000	2.220970000
6	0.830986000	-5.952894000	1.798086000
7	-0.740902000	-1.605783000	0.809346000
7	2.515122000	0.301596000	-0.183371000
7	-1.494025000	-5.088259000	-0.780640000
1	-3.850751000	-0.819007000	0.627685000
7	-1.232603000	-0.823561000	-2.938167000
7	2.228328000	-3.945832000	2.006256000
6	-0.868920000	-7.543418000	1.041728000
6	1.361556000	-6.863807000	2.714389000
6	0.763332000	-8.125194000	2.806514000
6	-0.332598000	-8.416272000	1.982938000
6	-3.566255000	-2.423796000	-4.254726000
6	-3.714673000	-4.988690000	-2.976539000
6	-4.602722000	-4.655298000	-4.005503000
6	-4.509574000	-3.387975000	-4.595800000
6	1.997965000	2.690499000	-2.122254000
6	1.493468000	3.543689000	-3.098699000
6	-0.236206000	2.012669000	-3.790794000
6	0.415732000	3.241743000	-3.941613000
6	4.682047000	-2.414555000	3.190416000
6	5.668238000	-1.476418000	3.478635000
6	4.859300000	0.127054000	1.869630000
6	5.794583000	-0.232342000	2.846243000
16	-1.132273000	-10.098925000	2.169182000
16	-5.754552000	-2.958837000	-5.926642000
16	2.301328000	5.221400000	-3.293957000
8	-5.067963000	-1.897053000	-6.979408000
8	-6.437181000	-4.359510000	-6.460497000
8	-1.793603000	-10.537486000	0.727746000
8	-0.071643000	-11.087100000	2.951046000
8	8.209197000	-0.892056000	4.633691000
8	7.119633000	-3.533132000	4.849907000
8	3.881270000	5.108981000	-2.848511000
8	1.876527000	5.827075000	-4.765497000
1	-5.344520000	-5.357259000	-4.369692000
1	-3.749792000	-5.959156000	-2.495956000
1	-3.501402000	-1.471876000	-4.768552000

1	-1.689705000	-7.827427000	0.393681000
1	1.139951000	-8.887890000	3.478872000
1	2.215165000	-6.600034000	3.327384000
1	4.649532000	-3.384343000	3.672862000
1	4.924565000	1.075598000	1.349998000
1	6.625211000	0.413791000	3.107306000
1	2.862775000	2.948091000	-1.522236000
1	0.123142000	3.953685000	-4.705266000
1	-1.070255000	1.736694000	-4.424955000
16	6.918548000	-1.900708000	4.806330000
6	-7.100223000	-2.006086000	-4.991745000
1	-7.809212000	-1.679536000	-5.751171000
1	-7.549548000	-2.704783000	-4.287776000
1	-6.614266000	-1.166976000	-4.495622000
6	-2.575023000	-9.786094000	3.357432000
1	-3.098298000	-10.737651000	3.439237000
1	-2.140267000	-9.476923000	4.306665000
1	-3.196875000	-9.012195000	2.909682000
6	1.440534000	6.289136000	-1.985879000
1	1.926498000	7.262433000	-2.036104000S
1	0.389399000	6.343222000	-2.264856000
1	1.593592000	5.802618000	-1.023502000
6	6.049230000	-1.417296000	6.419125000
1	6.730515000	-1.707866000	7.217505000
1	5.896426000	-0.339734000	6.385497000
1	5.112941000	-1.972328000	6.455848000
1	-2.633395000	-0.138235000	-0.039704000
1	0.114623000	-2.062331000	3.257120000

^{2}[Pc**S**]

26	0.455952000	-2.358790000	-0.430282000
7	0.586852000	-0.619482000	-1.343958000
7	-1.062130000	-2.836144000	-1.589093000
7	2.012263000	-1.906374000	0.703663000
7	0.365389000	-4.122851000	0.456402000
6	1.537786000	0.341889000	-1.082899000
6	-1.727993000	-4.042894000	-1.567894000
6	-0.214989000	-0.182006000	-2.375509000
6	-1.604098000	-2.046799000	-2.578539000
6	2.732434000	-0.732538000	0.622994000
6	-0.531520000	-5.120288000	0.135218000
6	2.598160000	-2.720309000	1.649969000
6	1.206590000	-4.586676000	1.446430000
6	1.329898000	1.474770000	-1.981815000
6	-2.774377000	-4.032142000	-2.588089000
6	0.225725000	1.144466000	-2.799578000
6	-2.696444000	-2.771126000	-3.221837000
6	3.840167000	-0.780852000	1.573902000
6	-0.264690000	-6.288409000	0.970311000

6	3.753870000	-2.034668000	2.220970000
6	0.830986000	-5.952894000	1.798086000
7	2.515122000	0.301596000	-0.183371000
7	-1.494025000	-5.088259000	-0.780640000
7	-1.232603000	-0.823561000	-2.938167000
7	2.228328000	-3.945832000	2.006256000
6	-0.868920000	-7.543418000	1.041728000
6	1.361556000	-6.863807000	2.714389000
6	0.763332000	-8.125194000	2.806514000
6	-0.332598000	-8.416272000	1.982938000
6	-3.566255000	-2.423796000	-4.254726000
6	-3.714673000	-4.988690000	-2.976539000
6	-4.602722000	-4.655298000	-4.005503000
6	-4.509574000	-3.387975000	-4.595800000
6	1.997965000	2.690499000	-2.122254000
6	1.493468000	3.543689000	-3.098699000
6	-0.236206000	2.012669000	-3.790794000
6	0.415732000	3.241743000	-3.941613000
6	4.682047000	-2.414555000	3.190416000
6	5.668238000	-1.476418000	3.478635000
6	4.859300000	0.127054000	1.869630000
6	5.794583000	-0.232342000	2.846243000
16	-1.132273000	-10.098925000	2.169182000
16	-5.754552000	-2.958837000	-5.926642000
16	2.301328000	5.221400000	-3.293957000
8	-5.067963000	-1.897053000	-6.979408000
8	-6.437181000	-4.359510000	-6.460497000
8	-1.793603000	-10.537486000	0.727746000
8	-0.071643000	-11.087100000	2.951046000
8	8.209197000	-0.892056000	4.633691000
8	7.119633000	-3.533132000	4.849907000
8	3.881270000	5.108981000	-2.848511000
8	1.876527000	5.827075000	-4.765497000
1	-5.344520000	-5.357259000	-4.369692000
1	-3.749792000	-5.959156000	-2.495956000
1	-3.501402000	-1.471876000	-4.768552000
1	-1.689705000	-7.827427000	0.393681000
1	1.139951000	-8.887890000	3.478872000
1	2.215165000	-6.600034000	3.327384000
1	4.649532000	-3.384343000	3.672862000
1	4.924565000	1.075598000	1.349998000
1	6.625211000	0.413791000	3.107306000
1	2.862775000	2.948091000	-1.522236000
1	0.123142000	3.953685000	-4.705266000
1	-1.070255000	1.736694000	-4.424955000
16	6.918548000	-1.900708000	4.806330000
6	-7.100223000	-2.006086000	-4.991745000
1	-7.809212000	-1.679536000	-5.751171000
1	-7.549548000	-2.704783000	-4.287776000

1	-6.614266000	-1.166976000	-4.495622000
6	-2.575023000	-9.786094000	3.357432000
1	-3.098298000	-10.737651000	3.439237000
1	-2.140267000	-9.476923000	4.306665000
1	-3.196875000	-9.012195000	2.909682000
6	1.440534000	6.289136000	-1.985879000
1	1.926498000	7.262433000	-2.036104000
1	0.389399000	6.343222000	-2.264856000
1	1.593592000	5.802618000	-1.023502000
6	6.049230000	-1.417296000	6.419125000
1	6.730515000	-1.707866000	7.217505000
1	5.896426000	-0.339734000	6.385497000
1	5.112941000	-1.972328000	6.455848000

^{2}[(Pc**H**)FeOOH]-N-$(NH_3)_2(NH_2)_2$

26	-1.913541000	-0.873745000	2.095995000
7	-3.215059000	-0.341537000	0.710473000
7	-1.063578000	0.900930000	1.956185000
7	-2.799942000	-2.635994000	2.279593000
7	-0.642914000	-1.398553000	3.521379000
6	-0.003991000	1.336091000	2.727177000
6	-3.264981000	0.879091000	0.068913000
6	-1.463547000	1.928998000	1.128513000
6	-3.931120000	-3.027001000	1.591182000
6	0.340477000	-0.579390000	4.040702000
6	-2.468222000	-3.624637000	3.185521000
6	-0.640394000	-2.588762000	4.219755000
6	-5.041030000	-0.358437000	-0.714924000
6	0.306060000	2.720394000	2.373728000
6	-4.403516000	0.894476000	-0.848277000
6	-0.616396000	3.095035000	1.371372000
6	-4.350517000	-4.344052000	2.069522000
6	1.021244000	-1.279254000	5.128298000
6	-3.433348000	-4.716693000	3.076978000
6	0.406993000	-2.546415000	5.238333000
26	0.380477000	-2.216516000	-0.394076000
7	0.782924000	-0.523315000	-1.319549000
7	-0.975281000	-2.616251000	-1.762721000
7	2.016182000	-1.986544000	0.677520000
7	0.254652000	-4.073838000	0.240320000
1	1.711110000	-0.637242000	-1.673796000
1	-1.516974000	-3.477173000	-1.838756000
1	0.139456000	-0.442706000	-2.080766000
1	-1.298138000	-1.978325000	-2.490192000
1	2.623740000	-1.167246000	0.682546000
1	-0.451460000	-4.513978000	-0.314371000
1	2.401958000	-2.662130000	1.337187000
1	1.147306000	-4.474758000	0.034329000
7	-0.757492000	-1.532026000	0.758318000

7	0.643439000	0.662988000	3.674017000
7	-4.597484000	-2.343192000	0.664684000
6	-4.269274000	-1.114707000	0.272233000
7	-1.470195000	-3.617807000	4.064796000
7	-2.458609000	1.920488000	0.245774000
6	-0.613245000	4.384046000	0.833538000
6	1.259946000	3.622214000	2.849992000
6	1.266471000	4.913553000	2.310976000
6	0.336774000	5.291742000	1.318245000
6	-4.900633000	1.870483000	-1.714376000
6	-6.060190000	1.571831000	-2.439655000
6	-6.705025000	0.324962000	-2.295549000
6	-6.199281000	-0.657215000	-1.434050000
6	-5.425448000	-5.168930000	1.731616000
6	-5.572529000	-6.371887000	2.431697000
6	-4.663459000	-6.737649000	3.447569000
6	-3.578990000	-5.915565000	3.778404000
6	0.813333000	-3.462751000	6.210674000
6	1.841827000	-3.081837000	7.079877000
6	2.447532000	-1.811062000	6.976747000
6	2.046607000	-0.896443000	5.995203000
1	-6.690081000	-1.616727000	-1.312955000
1	-7.620170000	0.134015000	-2.849289000
1	-6.475221000	2.310492000	-3.118567000
1	-4.401265000	2.828177000	-1.810401000
1	-6.123566000	-4.873757000	0.956403000
1	-6.401172000	-7.033023000	2.196879000
1	-4.823411000	-7.663212000	3.993814000
1	-2.878410000	-6.182969000	4.561923000
1	0.336562000	-4.433399000	6.286963000
1	2.176927000	-3.769515000	7.850374000
1	3.222951000	-1.533768000	7.685608000
1	2.500707000	0.084656000	5.910488000
1	1.966011000	3.323703000	3.616550000
1	1.993527000	5.639187000	2.662739000
1	0.349682000	6.310237000	0.939722000
1	-1.335251000	4.664859000	0.074458000
8	-2.987915000	-0.279086000	3.478391000
8	-3.861555000	0.906314000	3.096138000
1	-3.984983000	1.292540000	3.991657000
1	-0.032935000	-4.072033000	1.198073000
1	0.655547000	0.245696000	-0.693133000

^{2}[(Pc**H**)FeOOH]

26	-1.913541000	-0.873745000	2.095995000
7	-3.215059000	-0.341537000	0.710473000
7	-1.063578000	0.900930000	1.956185000
7	-2.799942000	-2.635994000	2.279593000
7	-0.642914000	-1.398553000	3.521379000

6	-0.003991000	1.336091000	2.727177000
6	-3.264981000	0.879091000	0.068913000
6	-1.463547000	1.928998000	1.128513000
6	-3.931120000	-3.027001000	1.591182000
6	0.340477000	-0.579390000	4.040702000
6	-2.468222000	-3.624637000	3.185521000
6	-0.640394000	-2.588762000	4.219755000
6	-5.041030000	-0.358437000	-0.714924000
6	0.306060000	2.720394000	2.373728000
6	-4.403516000	0.894476000	-0.848277000
6	-0.616396000	3.095035000	1.371372000
6	-4.350517000	-4.344052000	2.069522000
6	1.021244000	-1.279254000	5.128298000
6	-3.433348000	-4.716693000	3.076978000
6	0.406993000	-2.546415000	5.238333000
7	0.643439000	0.662988000	3.674017000
7	-4.597484000	-2.343192000	0.664684000
6	-4.269274000	-1.114707000	0.272233000
7	-1.470195000	-3.617807000	4.064796000
7	-2.458609000	1.920488000	0.245774000
6	-0.613245000	4.384046000	0.833538000
6	1.259946000	3.622214000	2.849992000
6	1.266471000	4.913553000	2.310976000
6	0.336774000	5.291742000	1.318245000
6	-4.900633000	1.870483000	-1.714376000
6	-6.060190000	1.571831000	-2.439655000
6	-6.705025000	0.324962000	-2.295549000
6	-6.199281000	-0.657215000	-1.434050000
6	-5.425448000	-5.168930000	1.731616000
6	-5.572529000	-6.371887000	2.431697000
6	-4.663459000	-6.737649000	3.447569000
6	-3.578990000	-5.915565000	3.778404000
6	0.813333000	-3.462751000	6.210674000
6	1.841827000	-3.081837000	7.079877000
6	2.447532000	-1.811062000	6.976747000
6	2.046607000	-0.896443000	5.995203000
1	-6.690081000	-1.616727000	-1.312955000
1	-7.620170000	0.134015000	-2.849289000
1	-6.475221000	2.310492000	-3.118567000
1	-4.401265000	2.828177000	-1.810401000
1	-6.123566000	-4.873757000	0.956403000
1	-6.401172000	-7.033023000	2.196879000
1	-4.823411000	-7.663212000	3.993814000
1	-2.878410000	-6.182969000	4.561923000
1	0.336562000	-4.433399000	6.286963000
1	2.176927000	-3.769515000	7.850374000
1	3.222951000	-1.533768000	7.685608000
1	2.500707000	0.084656000	5.910488000
1	1.966011000	3.323703000	3.616550000

1	1.993527000	5.639187000	2.662739000
1	0.349682000	6.310237000	0.939722000
1	-1.335251000	4.664859000	0.074458000
8	-2.987915000	-0.279086000	3.478391000
8	-3.861555000	0.906314000	3.096138000
1	-3.984983000	1.292540000	3.991657000

^{2}[Pc**H**]-N-$(NH_3)_2(NH_2)_2$

26	-1.913541000	-0.873745000	2.095995000
7	-3.215059000	-0.341537000	0.710473000
7	-1.063578000	0.900930000	1.956185000
7	-2.799942000	-2.635994000	2.279593000
7	-0.642914000	-1.398553000	3.521379000
6	-0.003991000	1.336091000	2.727177000
6	-3.264981000	0.879091000	0.068913000
6	-1.463547000	1.928998000	1.128513000
6	-3.931120000	-3.027001000	1.591182000
6	0.340477000	-0.579390000	4.040702000
6	-2.468222000	-3.624637000	3.185521000
6	-0.640394000	-2.588762000	4.219755000
6	-5.041030000	-0.358437000	-0.714924000
6	0.306060000	2.720394000	2.373728000
6	-4.403516000	0.894476000	-0.848277000
6	-0.616396000	3.095035000	1.371372000
6	-4.350517000	-4.344052000	2.069522000
6	1.021244000	-1.279254000	5.128298000
6	-3.433348000	-4.716693000	3.076978000
6	0.406993000	-2.546415000	5.238333000
26	0.380477000	-2.216516000	-0.394076000
7	0.782924000	-0.523315000	-1.319549000
7	-0.975281000	-2.616251000	-1.762721000
7	2.016182000	-1.986544000	0.677520000
7	0.254652000	-4.073838000	0.240320000
1	1.711110000	-0.637242000	-1.673796000
1	-1.516974000	-3.477173000	-1.838756000
1	0.139456000	-0.442706000	-2.080766000
1	-1.298138000	-1.978325000	-2.490192000
1	2.623740000	-1.167246000	0.682546000
1	-0.451460000	-4.513978000	-0.314371000
1	2.401958000	-2.662130000	1.337187000
1	1.147306000	-4.474758000	0.034329000
7	-0.757492000	-1.532026000	0.758318000
7	0.643439000	0.662988000	3.674017000
7	-4.597484000	-2.343192000	0.664684000
6	-4.269274000	-1.114707000	0.272233000
7	-1.470195000	-3.617807000	4.064796000
7	-2.458609000	1.920488000	0.245774000
6	-0.613245000	4.384046000	0.833538000
6	1.259946000	3.622214000	2.849992000

6	1.266471000	4.913553000	2.310976000
6	0.336774000	5.291742000	1.318245000
6	-4.900633000	1.870483000	-1.714376000
6	-6.060190000	1.571831000	-2.439655000
6	-6.705025000	0.324962000	-2.295549000
6	-6.199281000	-0.657215000	-1.434050000
6	-5.425448000	-5.168930000	1.731616000
6	-5.572529000	-6.371887000	2.431697000
6	-4.663459000	-6.737649000	3.447569000
6	-3.578990000	-5.915565000	3.778404000
6	0.813333000	-3.462751000	6.210674000
6	1.841827000	-3.081837000	7.079877000
6	2.447532000	-1.811062000	6.976747000
6	2.046607000	-0.896443000	5.995203000
1	-6.690081000	-1.616727000	-1.312955000
1	-7.620170000	0.134015000	-2.849289000
1	-6.475221000	2.310492000	-3.118567000
1	-4.401265000	2.828177000	-1.810401000
1	-6.123566000	-4.873757000	0.956403000
1	-6.401172000	-7.033023000	2.196879000
1	-4.823411000	-7.663212000	3.993814000
1	-2.878410000	-6.182969000	4.561923000
1	0.336562000	-4.433399000	6.286963000
1	2.176927000	-3.769515000	7.850374000
1	3.222951000	-1.533768000	7.685608000
1	2.500707000	0.084656000	5.910488000
1	1.966011000	3.323703000	3.616550000
1	1.993527000	5.639187000	2.662739000
1	0.349682000	6.310237000	0.939722000
1	-1.335251000	4.664859000	0.074458000
1	-0.032935000	-4.072033000	1.198073000
1	0.655547000	0.245696000	-0.693133000

^{2}[Pc**H**]

26	-1.913541000	-0.873745000	2.095995000
7	-3.215059000	-0.341537000	0.710473000
7	-1.063578000	0.900930000	1.956185000
7	-2.799942000	-2.635994000	2.279593000
7	-0.642914000	-1.398553000	3.521379000
6	-0.003991000	1.336091000	2.727177000
6	-3.264981000	0.879091000	0.068913000
6	-1.463547000	1.928998000	1.128513000
6	-3.931120000	-3.027001000	1.591182000
6	0.340477000	-0.579390000	4.040702000
6	-2.468222000	-3.624637000	3.185521000
6	-0.640394000	-2.588762000	4.219755000
6	-5.041030000	-0.358437000	-0.714924000
6	0.306060000	2.720394000	2.373728000
6	-4.403516000	0.894476000	-0.848277000

6	-0.616396000	3.095035000	1.371372000
6	-4.350517000	-4.344052000	2.069522000
6	1.021244000	-1.279254000	5.128298000
6	-3.433348000	-4.716693000	3.076978000
6	0.406993000	-2.546415000	5.238333000
7	0.643439000	0.662988000	3.674017000
7	-4.597484000	-2.343192000	0.664684000
6	-4.269274000	-1.114707000	0.272233000
7	-1.470195000	-3.617807000	4.064796000
7	-2.458609000	1.920488000	0.245774000
6	-0.613245000	4.384046000	0.833538000
6	1.259946000	3.622214000	2.849992000
6	1.266471000	4.913553000	2.310976000
6	0.336774000	5.291742000	1.318245000
6	-4.900633000	1.870483000	-1.714376000
6	-6.060190000	1.571831000	-2.439655000
6	-6.705025000	0.324962000	-2.295549000
6	-6.199281000	-0.657215000	-1.434050000
6	-5.425448000	-5.168930000	1.731616000
6	-5.572529000	-6.371887000	2.431697000
6	-4.663459000	-6.737649000	3.447569000
6	-3.578990000	-5.915565000	3.778404000
6	0.813333000	-3.462751000	6.210674000
6	1.841827000	-3.081837000	7.079877000
6	2.447532000	-1.811062000	6.976747000
6	2.046607000	-0.896443000	5.995203000
1	-6.690081000	-1.616727000	-1.312955000
1	-7.620170000	0.134015000	-2.849289000
1	-6.475221000	2.310492000	-3.118567000
1	-4.401265000	2.828177000	-1.810401000
1	-6.123566000	-4.873757000	0.956403000
1	-6.401172000	-7.033023000	2.196879000
1	-4.823411000	-7.663212000	3.993814000
1	-2.878410000	-6.182969000	4.561923000
1	0.336562000	-4.433399000	6.286963000
1	2.176927000	-3.769515000	7.850374000
1	3.222951000	-1.533768000	7.685608000
1	2.500707000	0.084656000	5.910488000
1	1.966011000	3.323703000	3.616550000
1	1.993527000	5.639187000	2.662739000
1	0.349682000	6.310237000	0.939722000
1	-1.335251000	4.664859000	0.074458000

^{2}[**9-OOH**]$_{A}^{-}$

26	-1.913541000	-0.873745000	2.095995000
7	-3.215059000	-0.341537000	0.710473000
7	-1.063578000	0.900930000	1.956185000
7	-2.799942000	-2.635994000	2.279593000
7	-0.642914000	-1.398553000	3.521379000

6	-0.003991000	1.336091000	2.727177000
6	-3.264981000	0.879091000	0.068913000
6	-1.463547000	1.928998000	1.128513000
6	-3.931120000	-3.027001000	1.591182000
6	0.340477000	-0.579390000	4.040702000
6	-2.468222000	-3.624637000	3.185521000
6	-0.640394000	-2.588762000	4.219755000
6	-5.041030000	-0.358437000	-0.714924000
6	0.306060000	2.720394000	2.373728000
6	-4.403516000	0.894476000	-0.848277000
6	-0.616396000	3.095035000	1.371372000
6	-4.350517000	-4.344052000	2.069522000
6	1.021244000	-1.279254000	5.128298000
6	-3.433348000	-4.716693000	3.076978000
6	0.406993000	-2.546415000	5.238333000
26	0.380477000	-2.216516000	-0.394076000
7	0.782924000	-0.523315000	-1.319549000
7	-0.975281000	-2.616251000	-1.762721000
7	2.016182000	-1.986544000	0.677520000
7	0.254652000	-4.073838000	0.240320000
6	1.786186000	0.371629000	-0.983666000
6	-1.711625000	-3.786535000	-1.866078000
6	0.079151000	0.010553000	-2.389067000
6	-1.414175000	-1.749051000	-2.751649000
6	2.842821000	-0.871813000	0.684358000
6	-0.674587000	-5.017574000	-0.174102000
6	2.540644000	-2.905001000	1.574335000
6	1.055564000	-4.665389000	1.204978000
6	1.713410000	1.536052000	-1.856987000
6	-2.665382000	-3.670892000	-2.963425000
6	0.646689000	1.305107000	-2.753094000
6	-2.486848000	-2.381239000	-3.510875000
6	3.941526000	-1.079891000	1.620357000
6	-0.473147000	-6.257980000	0.567224000
6	3.743099000	-2.356795000	2.187675000
6	0.626234000	-6.040518000	1.425786000
7	-0.757492000	-1.532026000	0.758318000
7	0.643439000	0.662988000	3.674017000
7	-4.597484000	-2.343192000	0.664684000
7	2.726609000	0.219160000	-0.060578000
7	-1.584479000	-4.884333000	-1.130138000
6	-4.269274000	-1.114707000	0.272233000
7	-1.470195000	-3.617807000	4.064796000
7	-0.938933000	-0.543028000	-3.036135000
7	-2.458609000	1.920488000	0.245774000
7	2.095740000	-4.127448000	1.827642000
6	-1.127269000	-7.491648000	0.525009000
6	1.130760000	-7.053638000	2.245416000
6	0.493348000	-8.297501000	2.212340000

6	-0.620400000	-8.466367000	1.376034000
6	-3.256750000	-1.936149000	-4.587193000
6	-3.594680000	-4.570935000	-3.493756000
6	-4.371916000	-4.144648000	-4.575685000
6	-4.193502000	-2.842983000	-5.067515000
6	2.481286000	2.699851000	-1.922579000
6	2.118964000	3.599637000	-2.917114000
6	0.332492000	2.216658000	-3.765909000
6	1.092294000	3.388571000	-3.849607000
6	4.634099000	-2.883477000	3.123597000
6	5.708901000	-2.066911000	3.450895000
6	5.051445000	-0.296314000	1.949692000
6	5.957582000	-0.807807000	2.884469000
6	-0.613245000	4.384046000	0.833538000
6	1.259946000	3.622214000	2.849992000
6	1.266471000	4.913553000	2.310976000
6	0.336774000	5.291742000	1.318245000
6	-4.900633000	1.870483000	-1.714376000
6	-6.060190000	1.571831000	-2.439655000
6	-6.705025000	0.324962000	-2.295549000
6	-6.199281000	-0.657215000	-1.434050000
6	-5.425448000	-5.168930000	1.731616000
6	-5.572529000	-6.371887000	2.431697000
6	-4.663459000	-6.737649000	3.447569000
6	-3.578990000	-5.915565000	3.778404000
6	0.813333000	-3.462751000	6.210674000
6	1.841827000	-3.081837000	7.079877000
6	2.447532000	-1.811062000	6.976747000
6	2.046607000	-0.896443000	5.995203000
16	-1.487003000	-10.125273000	1.404166000
16	-5.299805000	-2.274538000	-6.465433000
16	3.069211000	5.210691000	-3.001172000
16	6.911259000	-2.705756000	4.735701000
8	-4.643964000	-0.913286000	-7.114215000
8	-5.664336000	-3.573921000	-7.405978000
8	-2.467640000	-10.215613000	0.087582000
8	-0.367687000	-11.291136000	1.710283000
8	8.399221000	-2.082046000	4.416008000
8	6.685946000	-4.327903000	4.882777000
8	4.450501000	5.036061000	-2.125940000
8	3.114213000	5.716145000	-4.565716000
1	-5.086482000	-4.806964000	-5.052193000
1	-3.700368000	-5.567490000	-3.081227000
1	-3.136460000	-0.954900000	-5.030863000
1	-1.966605000	-7.691146000	-0.130823000
1	0.859221000	-9.134303000	2.797052000
1	1.994447000	-6.878427000	2.875517000
1	4.513119000	-3.867269000	3.560609000
1	5.207208000	0.669424000	1.483808000

1	6.856709000	-0.264929000	3.154698000
1	3.310167000	2.895689000	-1.252906000
1	0.917597000	4.119000000	-4.632103000
1	-0.469797000	2.014468000	-4.465791000
1	-6.690081000	-1.616727000	-1.312955000
1	-7.620170000	0.134015000	-2.849289000
1	-6.475221000	2.310492000	-3.118567000
1	-4.401265000	2.828177000	-1.810401000
1	-6.123566000	-4.873757000	0.956403000
1	-6.401172000	-7.033023000	2.196879000
1	-4.823411000	-7.663212000	3.993814000
1	-2.878410000	-6.182969000	4.561923000
1	0.336562000	-4.433399000	6.286963000
1	2.176927000	-3.769515000	7.850374000
1	3.222951000	-1.533768000	7.685608000
1	2.500707000	0.084656000	5.910488000
1	1.966011000	3.323703000	3.616550000
1	1.993527000	5.639187000	2.662739000
1	0.349682000	6.310237000	0.939722000
1	-1.335251000	4.664859000	0.074458000
8	-2.987915000	-0.279086000	3.478391000
6	1.972721000	6.433085000	-2.061376000
1	2.526366000	7.370775000	-2.047531000
1	1.042083000	6.518698000	-2.620117000
1	1.824141000	6.024899000	-1.062955000
6	-6.899286000	-1.757315000	-5.596548000
1	-7.546793000	-1.370116000	-6.381878000
1	-7.311975000	-2.652610000	-5.134026000
1	-6.633086000	-0.995383000	-4.865644000
6	-2.611120000	-10.029036000	2.923350000
1	-3.162210000	-10.968140000	2.938954000
1	-1.962761000	-9.923917000	3.791759000
1	-3.263237000	-9.168602000	2.782251000
6	6.321722000	-1.915553000	6.351033000
1	6.971744000	-2.317119000	7.126963000
1	6.440616000	-0.839147000	6.238309000
1	5.282331000	-2.208805000	6.488941000
8	-3.861555000	0.906314000	3.096138000
1	-3.984983000	1.292540000	3.991657000

^{2}[**9-OOH**]$_{B}^{-}$

26	-1.778414000	-0.982293000	2.069019000
7	-3.105838000	-0.186639000	0.840327000
7	-0.899348000	0.772705000	2.212246000
7	-2.920931000	-2.574795000	2.229266000
7	-0.714281000	-1.616104000	3.594463000
6	0.179436000	1.082643000	3.028209000
6	-3.040165000	1.078807000	0.277093000
6	-1.186164000	1.895336000	1.447519000

6	-4.055182000	-2.848657000	1.476128000
6	0.325450000	-0.931181000	4.208807000
6	-2.696263000	-3.661315000	3.062679000
6	-0.827074000	-2.853597000	4.212874000
6	-4.875215000	0.049827000	-0.650979000
6	0.604826000	2.455209000	2.778387000
6	-4.137580000	1.252518000	-0.668456000
6	-0.258810000	2.968468000	1.788013000
6	-4.578086000	-4.160962000	1.838836000
6	0.901044000	-1.759106000	5.262606000
6	-3.726904000	-4.669137000	2.842260000
6	0.181412000	-2.972309000	5.259404000
26	0.455952000	-2.358790000	-0.430282000
7	0.586852000	-0.619482000	-1.343958000
7	-1.062130000	-2.836144000	-1.589093000
7	2.012263000	-1.906374000	0.703663000
7	0.365389000	-4.122851000	0.456402000
6	1.537786000	0.341889000	-1.082899000
6	-1.727993000	-4.042894000	-1.567894000
6	-0.214989000	-0.182006000	-2.375509000
6	-1.604098000	-2.046799000	-2.578539000
6	2.732434000	-0.732538000	0.622994000
6	-0.531520000	-5.120288000	0.135218000
6	2.598160000	-2.720309000	1.649969000
6	1.206590000	-4.586676000	1.446430000
6	1.329898000	1.474770000	-1.981815000
6	-2.774377000	-4.032142000	-2.588089000
6	0.225725000	1.144466000	-2.799578000
6	-2.696444000	-2.771126000	-3.221837000
6	3.840167000	-0.780852000	1.573902000
6	-0.264690000	-6.288409000	0.970311000
6	3.753870000	-2.034668000	2.220970000
6	0.830986000	-5.952894000	1.798086000
7	-0.740902000	-1.605783000	0.809346000
7	0.745983000	0.299825000	3.940031000
7	-4.637268000	-2.057567000	0.581918000
7	2.515122000	0.301596000	-0.183371000
7	-1.494025000	-5.088259000	-0.780640000
6	-4.205276000	-0.833889000	0.296828000
8	1.656132000	-3.130891000	-1.609960000
7	-1.729177000	-3.796094000	3.963257000
7	-1.232603000	-0.823561000	-2.938167000
7	-2.156210000	2.032350000	0.550570000
7	2.228328000	-3.945832000	2.006256000
6	-0.868920000	-7.543418000	1.041728000
6	1.361556000	-6.863807000	2.714389000
6	0.763332000	-8.125194000	2.806514000
6	-0.332598000	-8.416272000	1.982938000
6	-3.566255000	-2.423796000	-4.254726000

6	-3.714673000	-4.988690000	-2.976539000
6	-4.602722000	-4.655298000	-4.005503000
6	-4.509574000	-3.387975000	-4.595800000
6	1.997965000	2.690499000	-2.122254000
6	1.493468000	3.543689000	-3.098699000
6	-0.236206000	2.012669000	-3.790794000
6	0.415732000	3.241743000	-3.941613000
6	4.682047000	-2.414555000	3.190416000
6	5.668238000	-1.476418000	3.478635000
6	4.859300000	0.127054000	1.869630000
6	5.794583000	-0.232342000	2.846243000
6	-0.139564000	4.285449000	1.338395000
6	1.615726000	3.242679000	3.334023000
6	1.742167000	4.559128000	2.879349000
6	0.871389000	5.076254000	1.896181000
6	-4.522143000	2.324002000	-1.477392000
6	-5.665289000	2.168095000	-2.267907000
6	-6.409085000	0.968861000	-2.243062000
6	-6.021492000	-0.105102000	-1.433672000
6	-5.689168000	-4.884574000	1.399596000
6	-5.933874000	-6.128025000	1.990644000
6	-5.088864000	-6.631409000	3.003017000
6	-3.973718000	-5.907312000	3.439474000
6	0.475440000	-3.990108000	6.169431000
6	1.504282000	-3.765906000	7.089510000
6	2.215812000	-2.546960000	7.101874000
6	1.923057000	-1.529541000	6.186735000
16	-1.132273000	-10.098925000	2.169182000
16	-5.754552000	-2.958837000	-5.926642000
16	2.301328000	5.221400000	-3.293957000
8	-5.067963000	-1.897053000	-6.979408000
8	-6.437181000	-4.359510000	-6.460497000
8	-1.793603000	-10.537486000	0.727746000
8	-0.071643000	-11.087100000	2.951046000
8	8.209197000	-0.892056000	4.633691000
8	7.119633000	-3.533132000	4.849907000
8	3.881270000	5.108981000	-2.848511000
8	1.876527000	5.827075000	-4.765497000
1	-5.344520000	-5.357259000	-4.369692000
1	-3.749792000	-5.959156000	-2.495956000
1	-3.501402000	-1.471876000	-4.768552000
1	-1.689705000	-7.827427000	0.393681000
1	1.139951000	-8.887890000	3.478872000
1	2.215165000	-6.600034000	3.327384000
1	4.649532000	-3.384343000	3.672862000
1	4.924565000	1.075598000	1.349998000
1	6.625211000	0.413791000	3.107306000
1	2.862775000	2.948091000	-1.522236000
1	0.123142000	3.953685000	-4.705266000

1	-1.070255000	1.736694000	-4.424955000
1	-6.589207000	-1.028451000	-1.400962000
1	-7.302036000	0.886995000	-2.856015000
1	-5.990234000	2.981173000	-2.909489000
1	-3.946875000	3.242783000	-1.486303000
1	-6.336404000	-4.486342000	0.626517000
1	-6.788833000	-6.715920000	1.671670000
1	-5.319415000	-7.591583000	3.455436000
1	-3.321841000	-6.279869000	4.221829000
1	-0.082443000	-4.919465000	6.156512000
1	1.758994000	-4.536697000	7.810090000
1	2.994341000	-2.396882000	7.844310000
1	2.458011000	-0.586310000	6.192471000
1	2.275734000	2.838344000	4.093004000
1	2.518486000	5.197312000	3.289654000
1	0.984981000	6.108842000	1.578949000
1	-0.819091000	4.674170000	0.588022000
16	6.918548000	-1.900708000	4.806330000
6	-7.100223000	-2.006086000	-4.991745000
1	-7.809212000	-1.679536000	-5.751171000
1	-7.549548000	-2.704783000	-4.287776000
1	-6.614266000	-1.166976000	-4.495622000
6	-2.575023000	-9.786094000	3.357432000
1	-3.098298000	-10.737651000	3.439237000
1	-2.140267000	-9.476923000	4.306665000
1	-3.196875000	-9.012195000	2.909682000
6	1.440534000	6.289136000	-1.985879000
1	1.926498000	7.262433000	-2.036104000
1	0.389399000	6.343222000	-2.264856000
1	1.593592000	5.802618000	-1.023502000
6	6.049230000	-1.417296000	6.419125000
1	6.730515000	-1.707866000	7.217505000
1	5.896426000	-0.339734000	6.385497000
1	5.112941000	-1.972328000	6.455848000
8	1.417216000	-2.725924000	-3.055502000
1	2.068834000	-3.322612000	-3.485586000
